# CD28 hinge used in chimeric antigen receptor (CAR) T-cells exhibits local structure and conformational exchange amidst global disorder

**DOI:** 10.1038/s42003-024-06770-w

**Published:** 2024-08-31

**Authors:** Varvara Folimonova, Xiang Chen, Hitendra Negi, Charles D. Schwieters, Jess Li, R. Andrew Byrd, Naomi Taylor, Philippe Youkharibache, Kylie J. Walters

**Affiliations:** 1 Protein Processing Section, Center for Structural Biology, Center for Cancer Research, National Cancer Institute, National Institutes of Health, Frederick, MD USA; 2 Computational Biomolecular Magnetic Resonance Core, National Institute of Diabetes and Digestive and Kidney Diseases, National Institutes of Health, Bethesda, MD USA; 3 Macromolecular NMR Section, Center for Structural Biology, Center for Cancer Research, National Cancer Institute, National Institutes of Health, Frederick, MD USA; 4 Pediatric Oncology Branch, Center for Cancer Research, National Cancer Institute, National Institutes of Health, Bethesda, MD USA; 5 Cancer Data Science Laboratory, Center for Cancer Research, National Cancer Institute, National Institutes of Health, Bethesda, MD USA

**Keywords:** Intrinsically disordered proteins, Cancer immunotherapy

## Abstract

T-cell therapies based on chimeric antigen receptor (CAR) targeting of a tumor-specific antigen offer hope for patients with relapsed or refractory cancers. CAR hinge and transmembrane regions link antigen recognition domains to intracellular signal transduction domains. Here, we apply biophysical methods to characterize the structure and dynamic properties of the CD28 CAR hinge (CD28H) used in an FDA-approved CD19 CAR for the treatment of B-lineage leukemia/lymphoma. By using nuclear Overhauser effect spectroscopy (NOESY), which detects even transiently occupied structural motifs, we observed otherwise elusive local structural elements amidst overall disorder in CD28H, including a conformational switch from a native β-strand to a 3_10_-helix and polyproline II helix-like structure. These local structural motifs contribute to an overall loosely formed extended geometry that could be captured by NOESY data. All FDA-approved CARs use prolines in the hinge region, which we find in CD28, and previously in CD8α, isomerize to promote structural plasticity and dynamics. These local structural elements may function in recognition and signaling events and constrain the spacing between the transmembrane and antigen recognition domains. Our study thus demonstrates a method for detecting local and transient structure within intrinsically disordered systems and moreover, our CD28H findings may inform future CAR design.

## Abstract (web summary)

A structural study of the CD28 hinge used in CAR T-cells reveals local structural elements amidst intrinsic disorder that may affect the spacing between the antigen recognition and transmembrane domains to impact CAR T-cell recognition and signaling.

## Introduction

Chimeric antigen receptors (CARs) have emerged as a transformative tool in the development of novel anti-tumor T cell-based therapies. Currently, six CAR T-cell therapies have been approved by the FDA to treat cancers, including B-cell lymphomas, B-cell acute lymphoblastic leukemia, and multiple myeloma^1^. CAR T-cell therapies have had much success in treating hematological malignancies by targeting CD19, CD20, and CD22 molecules expressed on B-lineage leukemias and lymphomas^2–4^ and a CAR T-cell therapy directed against B-cell maturation antigen (BCMA) has been developed to treat multiple myeloma^5,6^. Despite these successes, CAR T-cell therapy treatments face ongoing challenges, including antigen loss, toxicity, and unmet needs in solid tumors^7,8^. Implementations of CAR therapies continue to be developed, including bi-specific^9^ and tri-specific CARs against CD19, CD20, and CD22^10,11^.

**Fig. 1:**
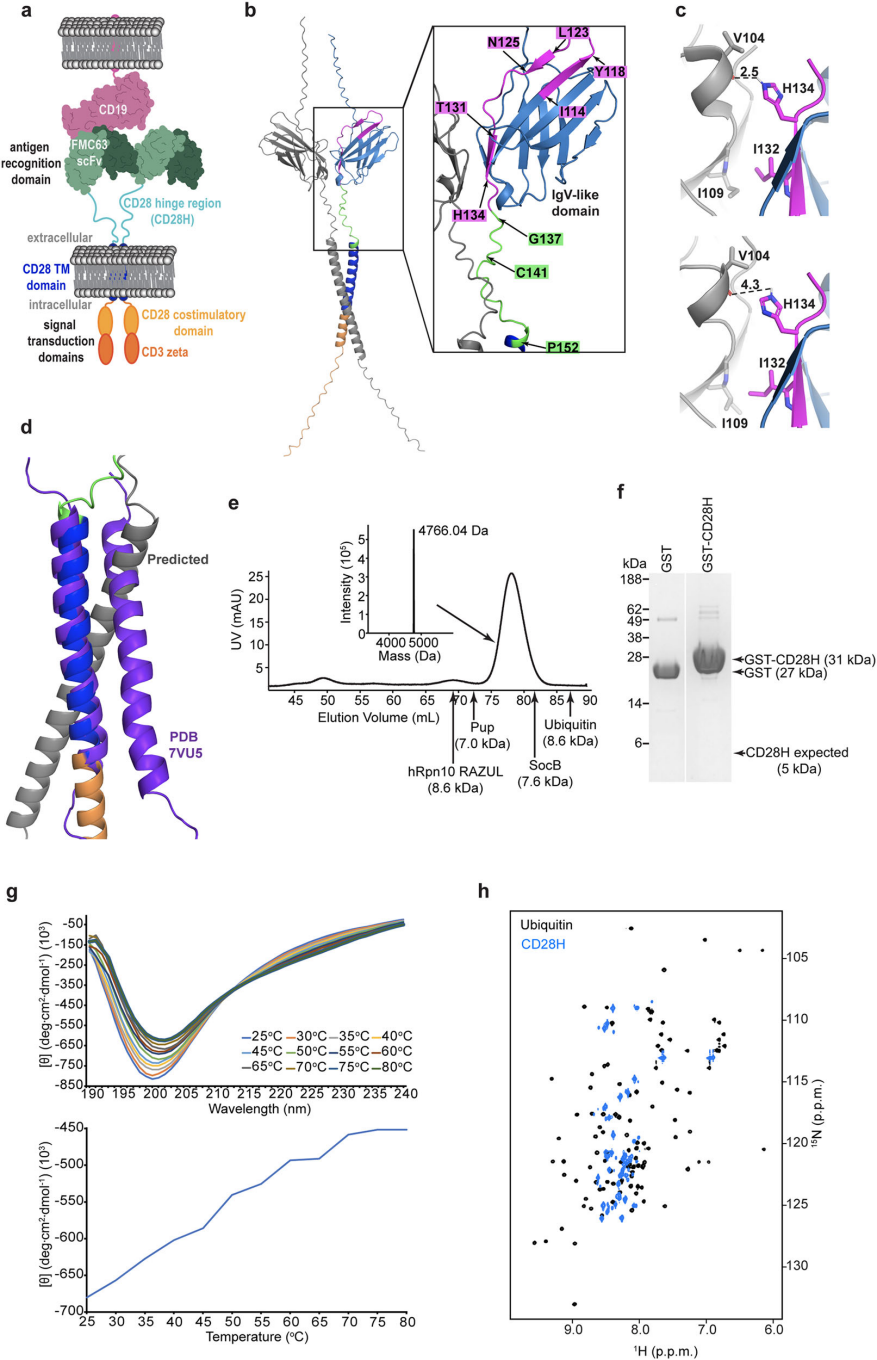
CD28H is globally disordered. **a** Illustration of dimeric FDA-approved CD19-based CAR axicabtagene ciloleucel highlighting the CD19-binding domain (green), CD28H (cyan) and transmembrane (TM) (indigo) regions, and intracellular signal transduction domains (shades of orange). **b** An AlphaFold 3 predicted structure visualized as a PyMOL cartoon for the CD28 protein dimer with one monomer in grey and the other monomer colored by domain to highlight the native intracellular region (orange), TM region (indigo), hinge (green), and IgV-like domain (blue) with the C-terminal end that is included in the CAR hinge in magenta. An expansion is included to highlight specific amino acids. **c** Comparison of the dimer interface of the CD28 IgV-like domain for experimental (top panel, PDB 1YJD) and predicted (bottom panel) structures in PyMOL. Distances are measured in Å. In both panels, the structures are colored with one monomer in grey and the other in blue, with the portion of the IgV-like domain that is included in the CAR hinge colored in magenta. **d** PyMOL cartoon structure of the CD28 transmembrane domain dimer (purple, PDB 7VU5) overlayed with the AlphaFold 3 predicted structure of the CD28 protein dimer colored as in **b**. **e** Chromatogram from size exclusion chromatography of ^15^N-labeled CD28H following injection onto an FPLC system equipped with a Superdex 75 pg column. The elution volumes for Pup, hRpn10 RAZUL, SocB, and ubiquitin are indicated on the chromatogram. The molecular weight of purified CD28H was tested and confirmed by LC-MS (top middle). **f** GST pull-down indicates that CD28H does not form oligomers. CD28H was incubated with glutathione beads prebound with GST (left, 27 kDa) or GST-tagged CD28H (right, 31 kDa), unbound proteins were removed by washing the beads, and those retained on the resin observed by SDS-PAGE. The expected position of CD28H (5 kDa) is indicated. **g** Circular dichroism spectra in the far-UV region at temperatures spanning from 25 to 80°C (top panel), and molar ellipticity at 196 nm plotted over temperature (bottom panel) for CD28H. **h** ^1^H, ^15^N HSQC spectrum of 500 μM ^15^N, ^13^C CD28H acquired at 800 MHz and 11°C alone (blue) and overlayed with ubiquitin (black) for comparison.

CAR constructs are engineered to target tumor-specific antigens, using single-chain variable fragments (scFv) or nanobodies. The CAR recognition domain is associated with intracellular signal transduction domains by an intervening hinge region and transmembrane domain and CARs can be monomeric or, as depicted by axicabtagene ciloleucel (Fig. 1a), dimeric^12,13^. Whereas structural information is available for the CAR antigen recognition, signal transduction and transmembrane domains, the hinge domain and the overall CAR structure has proven difficult to study due to their dynamic nature. Notably though, the hinge region is critical because it conditions the signaling threshold at which a CAR can respond to low antigen density tumors^14,15^ as well as its association with endogenous T cell receptor (TCR) components^16,17^.

We previously found that a CD22-targeting CAR incorporating a CD8α hinge exhibits greater cytotoxicity against CD22-low antigen density as compared to an equivalent CAR with a CD28 hinge (CD28H)^18^. This finding motivated us to apply biophysical methods, including nuclear magnetic resonance (NMR) spectroscopy, to study the structural and dynamic properties of the CD8α hinge region, identifying distinct conformational states undergoing dynamic exchange driven by proline isomerization^18^. Here, we expand these studies to CD28H and gain insights into the impact of this hinge on CAR activity. Our findings also have general implications for studies of structure within intrinsically disordered regions. In particular, we demonstrate that nuclear Overhauser effect spectroscopy (NOESY) is capable of detecting transient and otherwise elusive structural features within intrinsically disordered regions. By studying CD28H using NMR, and particularly through NOESY data, we observed local structure, including a sequence that adopts a helix in a globally disordered context despite forming a β-strand in its native context.

## Results

### CD28H exhibits global intrinsic disorder

We used AlphaFold 3^19^ to generate a model structure of the dimeric form of the CD28 protein expressed natively by human T-cells. Inputting two CD28 sequences yielded a structure with the IgV-like domain forming homodimers (Fig. 1b), similar to the experimentally determined crystal structure^20^. In the experimental structure, an intermolecular hydrogen bond forms between H134 and the backbone oxygen of V104 with intermolecular hydrophobic packing between I132 and I109 (Fig. 1c, upper panel). The predicted structure places these four residues at the dimeric interface, but further apart, disrupting the hydrogen bond between H134 and V104 (Fig. 1c, lower panel). The structure of the transmembrane domain was previously determined by NMR^21^; however, the predicted model differs from the experimental structure by an altered dimeric interface (Fig. 1d, purple versus grey)^21^. In the predicted structure, C141 of the hinge region is placed in proximity to form an intermolecular disulfide bond (Fig. 1b); no experimental structure exists for this region.

The CD28H in this study spans I114 – P152 (YESCARTA, Kite Pharma, Inc.), which includes residues I114 – K136 of the native IgV-like domain (Fig. 1b, magenta) and residues G137 – P152 from the native hinge region (Fig. 1b, green). Since residues I114 – Y118, L123 – D124, and T131 – H134 from the native IgV-like domain form β-strands^20^, we investigated how this region behaves in the CD28H context.

To study CD28H structurally, we generated an expression plasmid in *Escherichia coli* in frame with an N-terminal glutathione S-transferase (GST) tag followed by a PreScission protease cleavage site. Following its expression, we purified CD28H from *E. coli* by affinity chromatography followed by size exclusion chromatography (SEC) on an FPLC system (Fig. 1e and Supplementary Fig. 1). The resulting CD28H sample was validated by its expected molecular weight of 4,766.04 Da by liquid chromatography-mass spectrometry (LC-MS) (Fig. 1e, top middle); however, CD28H eluted earlier than expected by SEC for its molecular weight (Fig. 1e). For example, the larger 8.6 kDa protein ubiquitin elutes later than CD28H (Fig. 1e). Early elution from SEC is characteristic of intrinsic disorder, as we previously observed for prokaryotic ubiquitin-like protein (Pup)^22^, hRpn10 RAZUL^23^ and SocB^24^ (Fig. 1e). By contrast, early elution could also be caused by oligomerization. Of note, this experiment was performed in a buffer that contains 2 mM dithiothreitol (DTT), causing cysteine to be reduced. To test directly whether CD28H forms an oligomer, we conducted a GST pull-down assay in the presence of dithiothreitol with GST-tagged CD28H and CD28H (Fig. 1f). GST was also incubated with CD28H as a negative control. No bands were observed at the expected molecular weight for CD28H (5 kDa) (Fig. 1f and Supplementary Fig. 1), suggesting that CD28H does not bind to GST-tagged CD28H and that CD28H does not form oligomers in these conditions.

To evaluate further whether CD28H is intrinsically disordered, we tested whether it undergoes a phase transition during thermal denaturation by measuring molar ellipticity using circular dichroism (CD) (Fig. 1g). No cooperative protein unfolding was observed, consistent with the SEC data suggesting it to be intrinsically disordered and the GST pull-down assay indicating a monomer (Fig. 1f).

The amide backbone signals of structured proteins are dispersed in NMR spectra due to the divergent chemical environments of amino acids when in different secondary and tertiary structures. We therefore recorded a 2D ^1^H, ^15^N HSQC experiment on ^15^N-labeled CD28H to further evaluate the structural properties of CD28H. Whereas the amide signals of ubiquitin are dispersed in the ^1^H dimension (Fig. 1h, black), CD28H amide ^1^H signals are congested (Fig. 1h, blue), further indicating that overall, CD28H is intrinsically disordered.

### CD28H adopts distinct conformational states with proline cis/trans isomerization

**Fig. 2:**
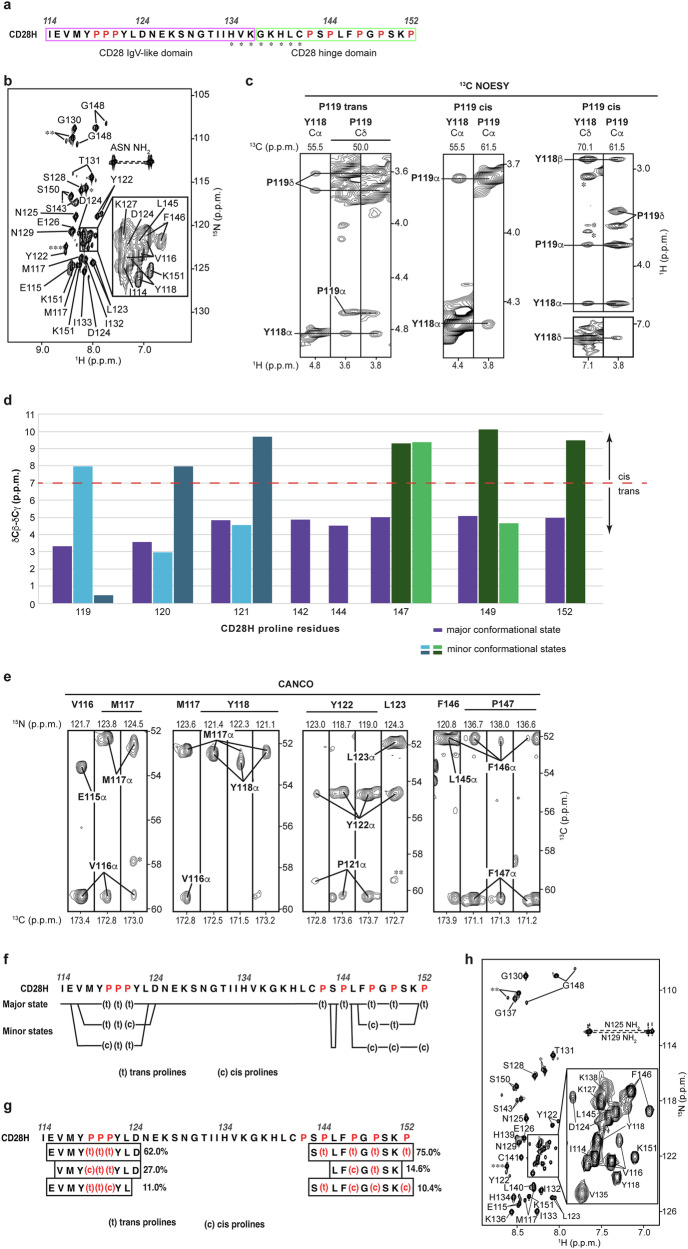
CD28H undergoes chemical exchange, with proline cis/trans isomerization. **a** Amino acid sequence of CD28H highlighting the IgV-like domain portion (I114 – K136, boxed in pink) and the hinge region (G137 – P152, boxed in green). Residues H134 – C141 (labeled with asterisks) do not appear in ^15^N-based NMR experiments acquired at 25°C, but at 11°C. **b** and **h** ^1^H, ^15^N HSQC spectrum of 500 μM ^15^N, ^13^C CD28H acquired at 800 MHz and 25°C (**b**) or 11°C (**h**), labeled with amino acid assignments. Signals from the non-native amino acid sequence LGS that remain following cleavage of the GST tag are labeled by three (***), two (**), or one (*) asterisk, respectively. **c** Selected regions from a ^13^C-dispersed NOESY experiment acquired at 850 MHz and 25°C (left and middle) or 800 MHz and 11°C (right) on a 600 μM sample of ^15^N, ^13^C CD28H, highlighting NOE interactions between proline Hδ and the Hα of the preceding residue within the major conformation of CD28H (left), and between Y118 Hα and the cis isomeric state of P119 (middle), and between Y118 Hδ and P119 Hα of the cis isomer of P119 (right). **d** Chemical shift differences of CD28H proline Cβ and Cγ atoms. **e** Strip plots of ^13^C, ^13^C planes from a 3D CANCO spectrum collected at 700 MHz on 450 μM ^15^N, ^13^C CD28H, showing where exchange signals converge with signals from the major conformational state. The signals labeled with asterisks are present in neighboring planes; the signal assigned as residue H134 is labeled with one asterisk (*), and the signal labeled with two asterisks (**) is assigned as residue M117. **f** Amino acid sequence of CD28H indicating for proline (red) the observed cis (c) or trans (t) isomeric states, and where the minor conformational states converge with the major state. **g** Amino acid sequence of CD28H and the percent populations of its conformational states, highlighting the observed cis (c) or trans (t) isomeric states of the prolines (red).

To gain detailed structural information for each amino acid within CD28H, we assigned the signals in the ^1^H, ^15^N HSQC spectrum to the individual amide atoms of CD28H by using modern NMR methods (Fig. 2a, b), as described in Materials and Methods. This analysis revealed the presence of multiple amide signals for certain amino acids (Fig. 2b). Multiple signals occur in NMR spectra when atoms exchange slowly (>100 ms time scale) between multiple conformational states. 3D NMR datasets, which were used to correlate sequential amino acids, also indicated multiple signals for Cα and Cβ atoms, such as in 3D HNCACB (Supplementary Fig. 2a, black and green) and CBCACONH (Supplementary Fig. 2a, purple) spectra. A 3D HACAN spectrum^25^ was used to assign proline rich regions and also contained multiple signals for individual proline atoms (Supplementary Fig. 2b, c).

CD28H has eight prolines (Fig. 2a) and to further characterize the distinct states of CD28H, we attempted to use NOESY data to assign the isomerization state as cis or trans for each proline signal. NOESY experiments detect distance-dependent signals between hydrogens that are within 5 Å of each other. A shorter distance between Hδ and the preceding amino acid’s Hα enables detection of a corresponding inter-residue NOE interaction in trans but not cis prolines. By contrast, cis prolines are uniquely characterized by their Hα atom having an NOE interaction with the Hα atom of the preceding amino acid. A ^13^C-dispersed NOESY spectrum acquired on ^13^C-labeled CD28H indicated NOE interactions for the trans isomer of all prolines except for P120 and P121, which were precluded from this analysis by overlapping signals for Cα and Hα atoms (Fig. 2c, left, for P119). Interactions were also observed between the Hα atoms of Y118 and P119, indicating that P119 also adopts the cis isomeric state (Fig. 2c, middle). Such inter-residue Hα NOEs were not observed for other proline residues; however, signal overlap and poor signal intensity limited this analysis. The chemical shift data of proline Cβ and Cγ atoms as obtained from 3D NMR data (HNCACB, CBCACONH, ^13^C-dispersed NOESY, CCH-TOCSY, and HCCH-TOCSY spectra) can also be used to evaluate the proline isomeric states^26,27^. We found the differences observed in Cβ and Cγ chemical shifts to be consistent with the isomeric states assigned by using NOEs (Fig. 2d).

By using a 3D CANCO experiment^28^ in combination with a ^15^N-dispersed NOESY experiment, we were able to assign NMR chemical shift values to the various conformational states of the residues in CD28H. Residues I114, N125 – P142, and P144 yielded a single peak each, indicating a single conformational state, while the spectrum indicated that other amino acids occupy up to three distinct conformational states (Fig. 2e, f, and Supplementary Table 1). Proline cis-trans isomerization can occur on a 400 ms time scale, which allows distinct NMR signals to be recorded for each state^29^. Careful evaluation of the 3D datasets indicated that except for P142 and P144, all prolines showed multiple sets of signals (Fig. 2f and Supplementary Fig. 3). For each proline, a set of signals associated with a trans isomeric state was observed, which was consistent with the NOESY analyses. Prolines 119, 121, and 149 exhibited an additional cis and trans isomeric state, while P152 exhibited only one additional set of cis isomer signals (Fig. 2f). Two additional sets of signals were observed for trans and cis isomers respectively for P120 and P147 (Fig. 2f).

To define the relative population of each conformational state, we integrated the signals of the 3D NMR spectra (including HACAN, HNCACB, and CANCO experiments) to obtain an averaged value (Fig. 2g). The major conformational state contains only trans proline isomers and represents 62% and 75% of the population respectively for the N-terminal stretch that includes ^119^PPP^121^ and the extreme C-terminal stretch. The lesser populated states contain different mixtures of cis and trans prolines. Only one conformational state is observable at I114 and for the region spanning N125 – P142 (Fig. 2f); however, this latter region includes H134 – C141, for which the amide signals are not observable at 25 °C due to conformational exchange at the so-called intermediate exchange time scale (Fig. 2a, b)^30^. We note that aliphatic signals were observed in this region at 25 °C and because of the inclusion of C141, we tested whether omission of DTT or inclusion of 20 μM ZnSO_4_ at induction causes the appearance of the amide signals. No changes were observed however with either of these alterations (Supplementary Fig. 4a, b). We tested and found however that amide signals for H134 – C141 do appear in spectra recorded at 11 °C (Fig. 2h). We therefore also collected ^15^N-dispersed and ^13^C-dispersed NOESY experiments at 11 °C and found greater signal-to-noise and additional NOE interactions throughout the sequence. In addition, the ^13^C-dispersed NOESY at 11 °C was optimized to record pyrrolidine-aromatic CH-π interactions and detected an NOE between Y118 Hδ and P119 Hα of the P119 cis isomer (Fig. 2c, right). Altogether, our data indicate that CD28H undergoes temperature-dependent conformational exchange that includes, but is not limited to, proline cis-trans isomerization.

### Local structural elements are formed in CD28H that diverge from native CD28

**Fig. 3:**
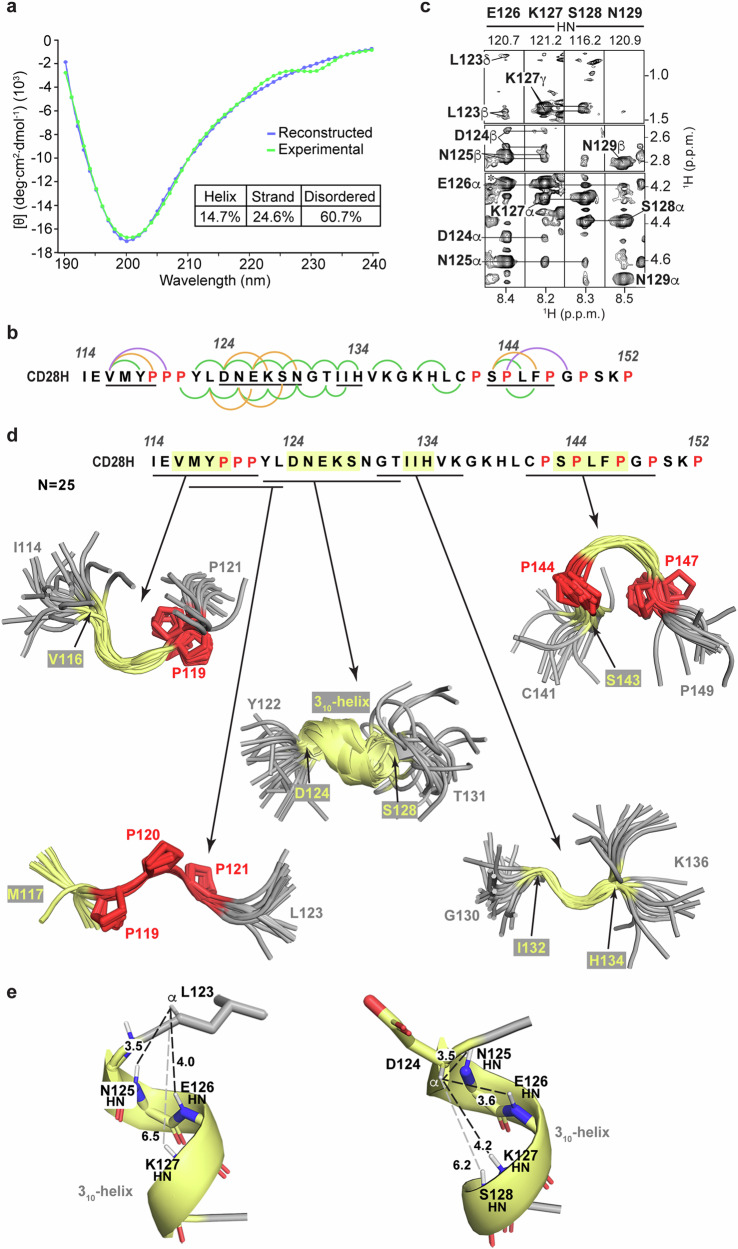
Local structure is detected amidst the global disorder of CD28H. **a** Far-UV circular dichroism spectra of experimental (green) and reconstructed (blue) data for CD28H from 190 to 240 nm wavelength. A table (bottom right) lists the percentage of predicted helical, β-strand, and disordered regions. **b** The CD28H sequence with regions exhibiting mid-range inter-residue NOEs underlined. i + 2, i + 3, and i + 4 NOEs are indicated by green, orange, and purple arcs, respectively. **c** ^1^H, ^15^N strip plots from an ^15^N-dispersed NOESY experiment recorded at 800 MHz and 11 °C on 400 μM ^15^N CD28H, showing i + 2 and i + 3 inter-residue NOE interactions for the region containing L123 – K127. An asterisk indicates an exchange peak for E126 Hα. **d** Aligned regions of CD28H structures calculated using NMR data, highlighting portions that exhibit local structure (yellow) and prolines (red). The CD28H sequence is included above the structural elements. **e** PyMOL cartoon structure of a region of CD28H highlighting interactions involving the 3_10_-helix formed by D124 – S128 with heavy sidechain atoms displayed as sticks and oxygen (with double or single valency indicated), nitrogen, and select hydrogen atoms colored red, indigo, and white, respectively. Distances between L123 Hα or D124 Hα and the amide group of residues i + 2, i + 3, or i + 4 are displayed in black or grey dashed lines. Distances are provided in Å.

To further investigate the structural properties of CD28H, we evaluated a CD spectrum recorded at 25 °C as in Fig. 1g for secondary structure by DichroWeb^31^. This analysis predicted 25% β-strand and 15% helicity, with 61% of the sequence predicted to be disordered (Fig. 3a). Although no long-range NOE interactions were observed in NOESY spectra recorded on CD28H, several medium-range inter-residue NOE correlations were observed at 11 °C (Fig. 3b). For example, within the region spanning L123 – N129, NOEs were detected from the Hβ and methyl groups of L123 to E126 HN, from D124 Hα and Hβ to K127 HN, and from N125 Hα to S128 HN (Fig. 3c). These NOEs suggest helicity for the region spanning L123 – N129, consistent with the CD prediction of 15% helicity (Fig. 3a).

We used our sparse NOESY data at 11 °C (Fig. 3b) to calculate structures for the major conformational state of CD28H by using Xplor-NIH 3.7^32^, with the structural statistics summarized in Table 1. The signals detected for the minor conformations were too weak for a comparable analysis or structure calculations. Backbone ϕ and ψ torsion angle restraints were also incorporated into the structure calculations – these restraints were defined by TALOS+ based on NMR chemical shift information at 11 °C. Although the calculated structures did not converge to a single solution overall, several short stretches formed loosely converged backbone structures (Fig. 3d). In the initial rounds of structure calculations, a 3_10_-helix was formed spanning D124 – S128, consistent with the NOE (Fig. 3b and c) and CD (Fig. 3a) data. Rather than observing an α-helical hydrogen bonding pattern between the carbonyl group of a residue i and the amide group of residue i + 4, CD28H exhibited a 3_10_-helix hydrogen bonding pattern between i and i + 3 residues (Fig. 3e). Based on these observations, long-range HNCO-COSY experiments^33,34^ were collected on CD28H to determine experimentally whether hydrogen bonds can be detected within D124 – S128. Only intra-residue hydrogen bonds involving sidechain atoms were observed, however. The detected signals were between the backbone amide atoms of D124 and N125 and their respective sidechain oxygen atoms (Supplementary Fig. 5). These two hydrogen bonds were added to the final structure calculations, however no other hydrogen bonds were included in the structure calculations.

**Table 1:** NMR and refinement statistics for CD28H

	Protein
**NMR distance and dihedral restraints**	
Distance restraints	
Total NOE	479
Intra-residue	266
Inter-residue	213
Sequential (|*i* – *j*| = 1)	159
Medium-range (|*i* – *j*| < 4)	54
Long-range (|*i* – *j*| > 5)	0
Hydrogen bonds	2
Total dihedral angle restraints	
ϕ	13
ψ	13
Structure statistics*	
Violations (mean and s.d.)	
Distance restraints (Å)	0.18 ± 0.06
Dihedral angle restraints (°)	1.11 ± 0.10
Max. dihedral angle violation (°)	<5
Max. distance restraint violation (Å)	<0.5
Deviations from idealized geometry	
Bond lengths (Å)	0.003 ± 0.000
Bond angles (°)	0.390 ± 0.028
Impropers (°)	0.212 ± 0.024
Average pairwise r.m.s. deviation** (Å)	
Heavy	1.10 ± 0.63
Backbone	2.35 ± 0.87

^*^Statistics for the 25 lowest energy structures without NOE, dihedral or torsion angle violations for CD28H (I114-P152).

^**^Pairwise r.m.s.d. for the 25 lowest energy structures for D124-S128.

**Fig. 4:**
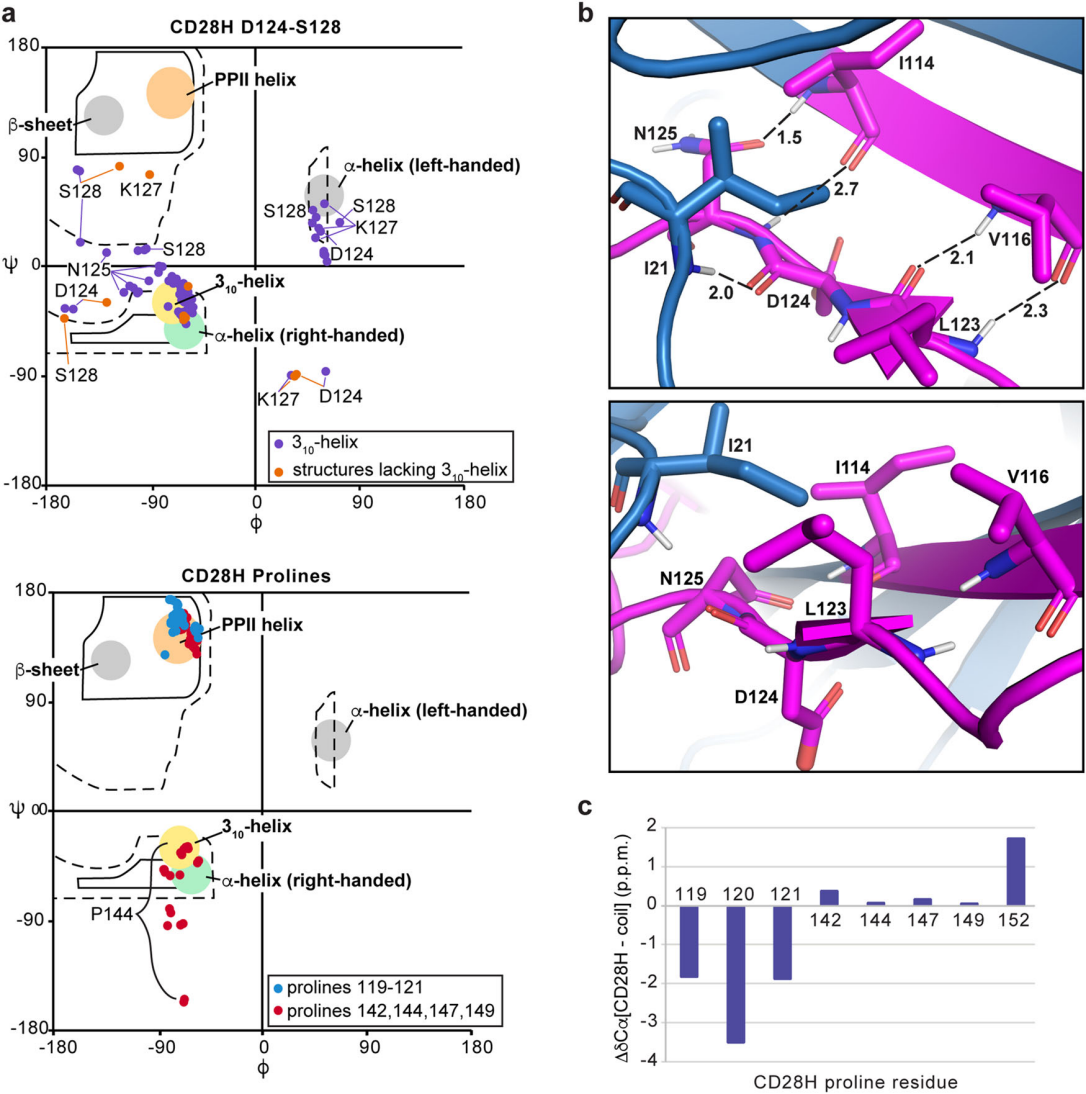
CD28H exhibits a 3_10_-helix and PPII helix-like structures. **a** Ramachandran plot of residues D124 – E126 (top panel, violet and orange), P119 – P121 (bottom panel, light blue), and P142, P144, P147, and P149 (bottom panel, red). In the plot of residues D124 – S128 (top panel), the torsion angles of structures that contain or lack a 3_10_-helix are colored orange or violet, respectively. Regions of the Ramachandran plot associated with a 3_10_-helix (yellow), α-helix (green), and PPII helix structure (orange) are indicated. **b** PyMOL cartoon structure depicting residues L123 – N125 and interacting residues I21, I114, and V116 from the crystal structure of the CD28 IgV-like domain (PDB 1YJD). The IgV-like domain (blue) is displayed highlighting the portion included in the CAR hinge (magenta), with backbone nitrogen, oxygen, and hydrogen amide atoms in indigo, red (with double or single valency indicated), and white, respectively. In the top panel, hydrogen bonding interactions are indicated with a dashed line and distances measured in Å. **c** Chemical shift index comparison for proline Cα atoms in CD28H compared to those in random coil structures.

Among the 25 calculated lowest energy structures, 23 form a 3_10_-helix that spans D124 – S128, and the ϕ and ψ torsion angles of all 25 structures are more consistent with a 3_10_-helix than an α-helix (Fig. 4a, top panel). The divergence from helicity for two of the structures may reflect transient exchange between a helical and disordered state, such that NOEs consistent with helicity are recorded when the helix is formed. As mentioned above, in the context of the intact IgV-like domain, L123 – N125 forms a β-strand^20^ stabilized by hydrogen bonds from L123 to V116, D124 to I21, and N125 to I114 (Fig. 4b, top panel). L123 is also incorporated into a hydrophobic patch that includes I21, I114, and V116, which cluster on one side of the β-sheet (Fig. 4b, bottom panel). Loss of the surrounding amino acids in CD28H causes a reconfiguration of the secondary structure to allow formation of the helix (Fig. 3e).

The structure calculations yielded a polyproline II (PPII) helix-like structure for the region spanning ^119^PPP^121^ (Fig. 3d), with characteristic torsion angles (Fig. 4a, bottom panel indicated in blue). By contrast, the ϕ and ψ angles of prolines 142, 144, 147, and 149 distribute to the multiple regions allowed for proline residues (Fig. 4a, bottom panel in red). This structure is supported by the Cα chemical shift values of ^119^PPP^121^, which are shifted downfield compared to the other prolines present in CD28H (Fig. 4c).

### NMR reveals local structure driven by interactions involving CD28H methyl groups

**Fig. 5:**
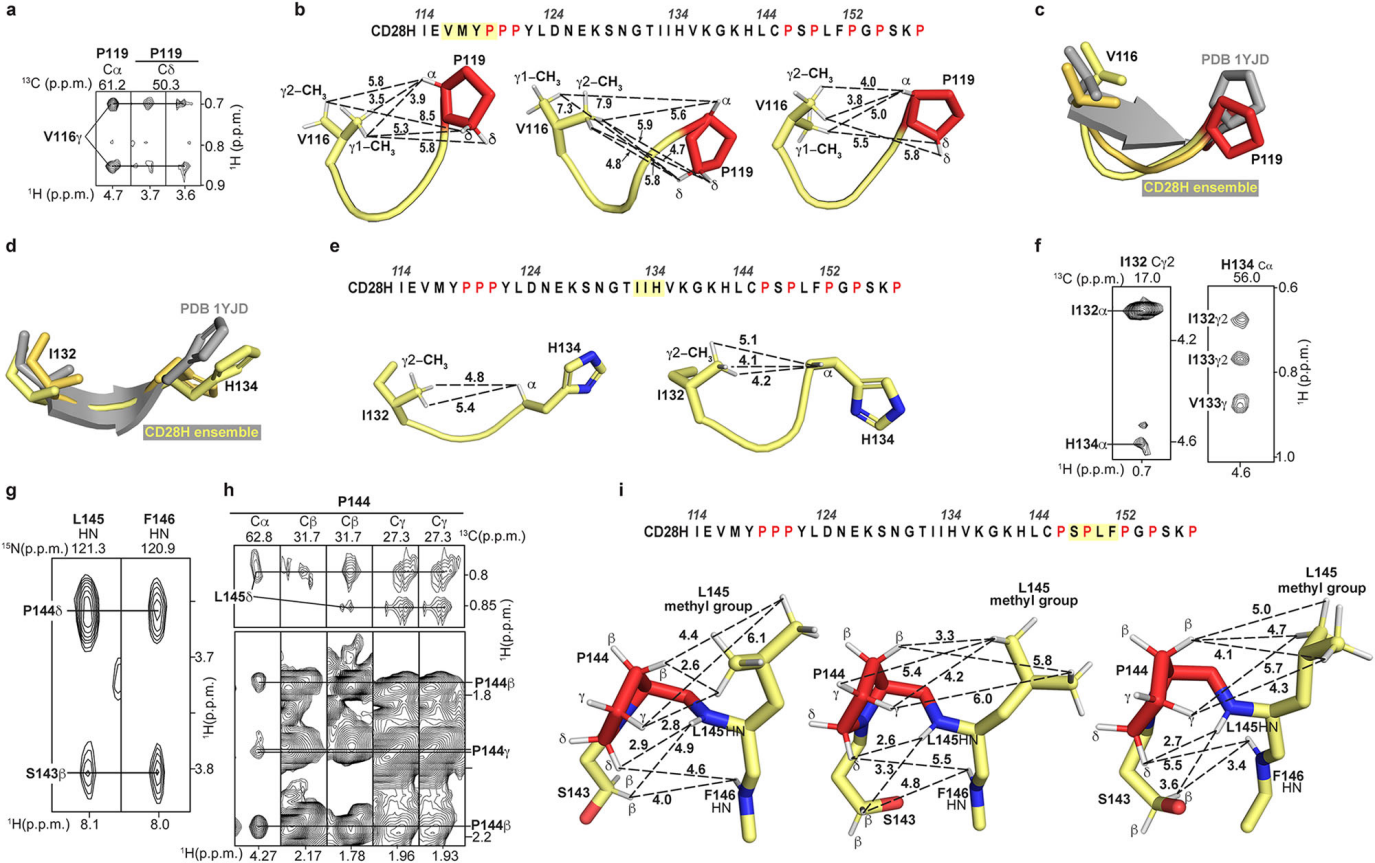
Methyl groups drive formation of local structural elements and show corresponding NOE interactions. **a**, **f**, **h** 2D ^1^H, ^13^C strip plots from a ^13^C-dispersed NOESY experiment and **g** ^1^H, ^15^N strip plots from a ^15^N-dispersed NOESY experiment showing i + 1, i + 2, or i + 3 inter-residue NOE interactions for CD28H. The ^13^C-dispersed and ^15^N-dispersed NOESY experiments were acquired at 800 MHz and 11°C on 600 μM ^13^C, ^15^N CD28H and 400 μM ^15^N CD28H respectively. **b, e, i** PyMOL cartoon structure of CD28H for select regions as labeled that exhibit i + 1, i + 2, or i + 3 inter-residue NOE interactions. The regions are highlighted in yellow along the sequence of CD28H. Distances are measured in Å. Prolines and oxygen atoms are colored in red; nitrogen and hydrogen atoms are indigo and white respectively. **c** and **d** PyMOL cartoon structures of residues (**c**) V116 – P119 and (**d**) I132 – H134 of the CD28 IgV-like domain in grey (PDB 1YJD) overlayed with two representative structures from the CD28H ensemble (yellow).

Three additional regions in CD28H show converged backbone structures spanning V116 – P119, I132 – H134, and S143 – P147 (Fig. 3d). Whereas I114 – Y118 forms a β-strand in the intact IgV domain as part of a β-sheet (Fig. 1b), V116 – P119 in CD28H is structurally defined by NOE interactions between the V116 methyl groups and P119 Hα and Hδ atoms (Fig. 5a). These interactions are weak but sufficient to restrict the overall backbone geometry of this local region and to induce a slight bend (Fig. 3d), providing van der Waals contacts for the methyl group (Fig. 5b). This bending is distinct from the native β-strand (Fig. 5c).

T131 – H134 forms a β-strand at the dimeric interface of the IgV-like domain in the intact CD28 dimeric protein (Fig. 1b). This region in CD28H mimics the native CD28 dimeric protein (Figs. 3d and 5d), with a bend that promotes van der Waals contacts to the I132 methyl group (Fig. 5e). This structure in CD28H is supported by weak NOE correlations between I132 and H134 (Fig. 5f). Variances in the sidechain conformations fit the sparse data, causing the distances between the I132 methyl groups and H134 Hα atom to differ (Fig. 5e) and for slight dispersion of the backbone geometry (Fig. 3d).

In the region spanning S143 – F146, NOE interactions were recorded from S143 Hβ to L145 and F146 HN atoms (Fig. 5g), and from the L145 methyl groups to P144 atoms (Fig. 5h). These data allow for some variance in sidechain conformations (Fig. 5i) but restrict the backbone geometry of S143 – F146 to an arched structure (Fig. 3d) with hydrophobic interactions between P144 and L145 (Fig. 5i). Altogether, these data indicate that CD28H has stretches of local structure, some of which diverge from the native CD28 dimeric protein, that is driven by van der Waals interactions, primarily involving methyl groups.

Axicabtagene ciloleucel CARs place the transmembrane domain of CD28 C-terminally adjacent to CD28H (Fig. 1a). To gain insight into the consistency of our structural findings in the context of the full CAR construct, we tested whether CD28H has affinity for a phospholipid membrane. We added 35 μM MSPΔH5 nanodiscs to 100 μM ^15^N-labeled CD28H and acquired ^1^H, ^15^N HSQC experiments to test for interaction. No spectral changes were induced for CD28H however by MSPΔH5 nanodiscs (Supplementary Fig. 6). This result suggests that CD28H is not likely to interact with the C-terminal transmembrane region of the intact CAR. Moreover, AlphaFold 3 did not predict an interaction to occur between CD28H and the N-terminally adjacent FMC63 antigen recognition domain. Altogether these data suggest that CD28H may act largely as an independent structural entity within the intact CAR.

### The N-terminal half of CD28H has a loosely converged extended global shape

Intrinsically disordered proteins are commonly described in terms of their radius of gyration, which has previously been found to increase linearly with sequence length in randomly coiled proteins^35^. The average radius of gyration of the 39-residue CD28H calculated ensemble representing the major state was computed to be 16.03 ± 2.87 Å, which falls within the range expected for an intrinsically disordered protein^35^. While CD28H has regions containing local structure, this protein sequence is overall globally disordered.

**Fig. 6:**
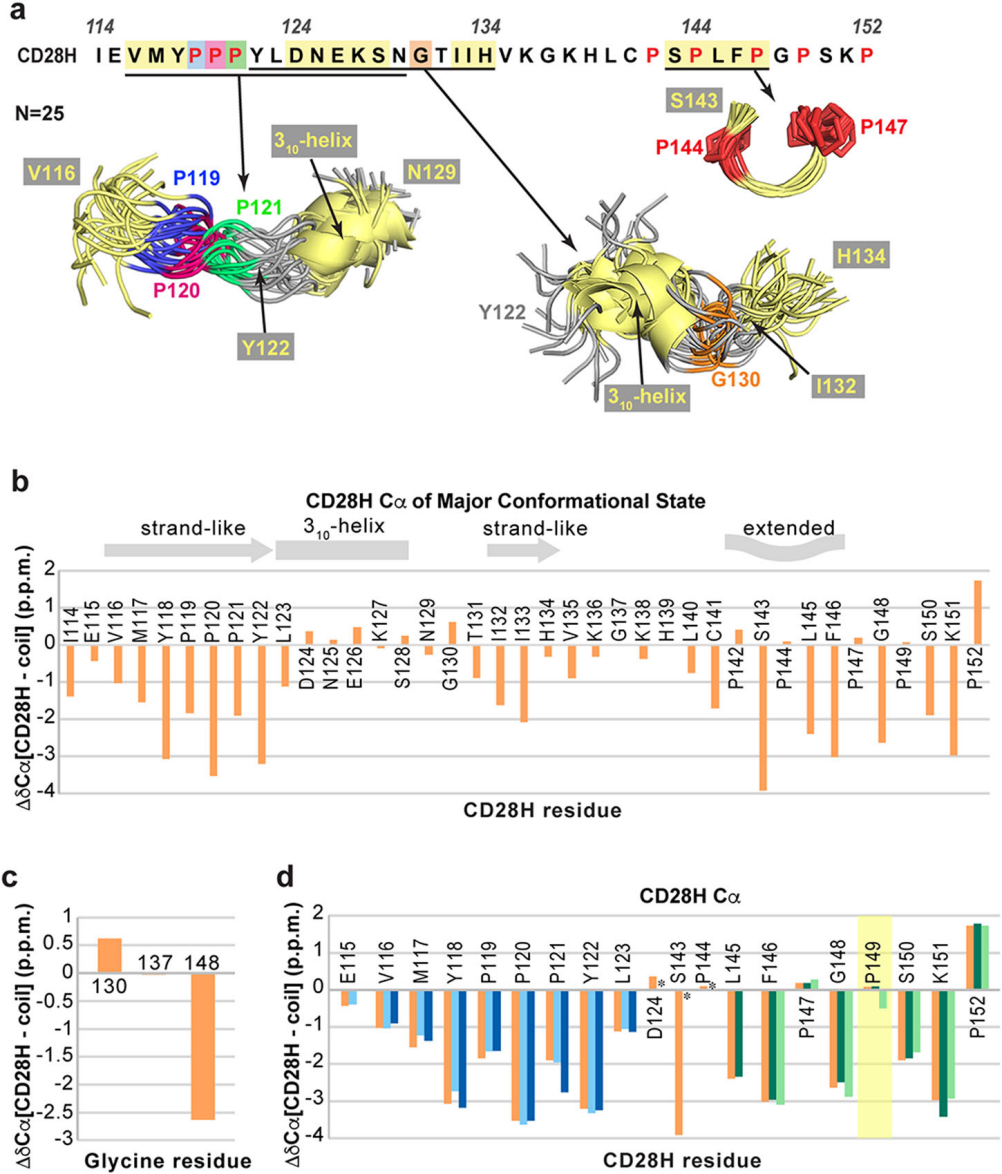
The CD28H ensemble adopts a loose global shape with local structure. **a** PyMOL cartoon depiction of aligned portions of 25 CD28H structures is calculated by using NMR data. Regions containing local structure are colored yellow with P119, P120, and P121 in blue, dark pink, and green, respectively. P142 and P144 are colored red and G130 is colored in orange. **b** Cα chemical shift index of the major conformational state of CD28H, with secondary structural features indicated above. **c** Cα chemical shift index comparison of CD28H glycine. **d** Cα chemical shift index of the major (orange) and minor (light blue, dark blue, dark green, and light green) conformational states of CD28H. The Cα chemical shifts of minor conformational states of D124, S143, and P144 were not determined and are labeled with an asterisk. P149 is highlighted in yellow.

Alignment of the whole CD28H sequence indicated overall disorder; however, two overlapping loosely ordered regions were apparent in the CD28H structural ensemble that were somewhat spatially restricted (Fig. 6a). The region spanning V116 – S128 contains small stretches of high convergence that includes the PPII helix-like structure and 3_10_-helix (Fig. 3d). When the broader region is aligned, the ensemble appears to have a slightly expanded global shape, driven by the ^119^PPP^121^ stretch (Fig. 6a). This extended shape is indicated by the comparison of Cα chemical shifts of CD28H to random coil values^36,37^, an analysis that predicts CD28H to have strand-like structure in the N-terminal portion spanning residues I114 – L123 (Fig. 6b), similar to the extended global shape observed in structure calculations (Fig. 6a). Downfield shifts support the helical structure of D124 – G130 (Fig. 6b), consistent with the calculated 3_10_-helical structure (Fig. 3d). Disorder was suggested by this analysis in the C-terminal portion of CD28H spanning residues T131 – P152 (Fig. 6b).

The region containing Y122 – H134 also exhibits a loose global shape, driven by the local structure of D124 – S128 and I132 – H134 (Fig. 6a). This larger portion features a bend at residue G130, where no medium-range inter-residue NOE interactions were observed (Fig. 6a). The bend observed in the region spanning Y122 – H134 forms due to G130 backbone ϕ (92.4^o^ ± 15.5^o^) and ψ (−0.3^o^ ± 12.7^o^) torsion angle restraints, which were derived by TALOS+ based on chemical shift information, including a downfield-shifted Cα signal (Fig. 6c). Altogether, our data indicate that although CD28H is disordered with only short regions of convergence, a loosely defined global shape exists in the N-terminal half, which in the native CD28 protein, is part of an IgV-like domain.

We further evaluated the minor conformational states of CD28H by similarly examining the chemical shift information. Overall, the values were similar to the dominant state (Fig. 6d, orange) except at residue P149 (Fig. 6d, highlighted in yellow), for which the Cα chemical shift value is shifted more upfield in one of the minor conformers (Fig. 6d, light green). This divergence suggests a different (trans) configuration for the backbone of P149 in a less populated state, however overall, the trends in Cα chemical shift values resemble the dominant state.

## Discussion

Our previous study, which found that the hinge region in the CD22 CAR conditions its cytotoxicity against CD22^low^ leukemia^18^ combined with findings that hinges influence signaling thresholds^14,15^ and association with endogenous cellular machinery^16,17^, motivated our structural characterization of the CD28H. The N-terminal half of CD28H was taken from the well-structured IgV-like domain with regions that form β-strands. We observed local structural features throughout the CD28H sequence; however, we find that when out of the full protein context, the β-strand region spanning D124 to E126 switches to a 3_10_-helix. This conversion can be rationalized by the loss of inter-strand interactions, such as with I21, which is missing from CD28H. Rather than such long-range interactions that can drive β-sheet formation, we detect shorter hydrophobic interactions involving methyl groups. Our study of CD28H indicates methyl groups to be a key driving force for local structure within CD28H. This finding is likely generally true for intrinsically disordered proteins. We note that NOE interactions are recorded only when atoms are within 6 Å of each other and signals are detected even for transiently populated structures. Therefore, it is plausible that the local structures we observe in CD28H are only transiently populated.

All hinges (CD28, CD8α, and IgG4) incorporated into the six FDA-approved CARs contain proline residues. Hinges from CD28 and CD8α are highly dynamic and form multiple conformational states in part due to proline isomerization^18^. The effect of prolines on CAR hinge plasticity and dynamics may be generalized to other hinge domains; however, future studies are needed to discern whether these effects can be promoted by other amino acids and/or combinations such as glycine, which also has the capacity to adopt a large range of backbone dihedral angles. For example, exchange broadening is observed for CD28H H134 – C141 at 25°C, a region that lacks proline but includes glycine.

We find considerable heterogeneity within CD28H, including proline isomerization, and such dynamics may contribute to CAR T-cell signaling. A limitation of our study is that we were unable to record NOE interactions for the lesser populated states; however, Cα chemical shift values were found to be similar in these states compared to the dominant population, suggesting similar structural features.

Intrinsically disordered proteins (IDPs) are very difficult to study by methods involving crystallography and microscopy, which are unable to capture dynamic structural features. Even NMR studies are stymied by the lack of chemical shift dispersion exhibited by IDPs, leading to potential signal overlap. In this study, chemical shift indices similarly struggled to capture the dynamic local structures present in CD28H (Fig. 6b). Nevertheless, NMR data is useful for studying the structure of IDPs, as demonstrated by a study of the structure of intrinsically disordered Tau in the context of varying phosphorylation states, leading to the discovery of helicity rather than a previously suggested β-turn structure^38,39^.

**Fig. 7:**
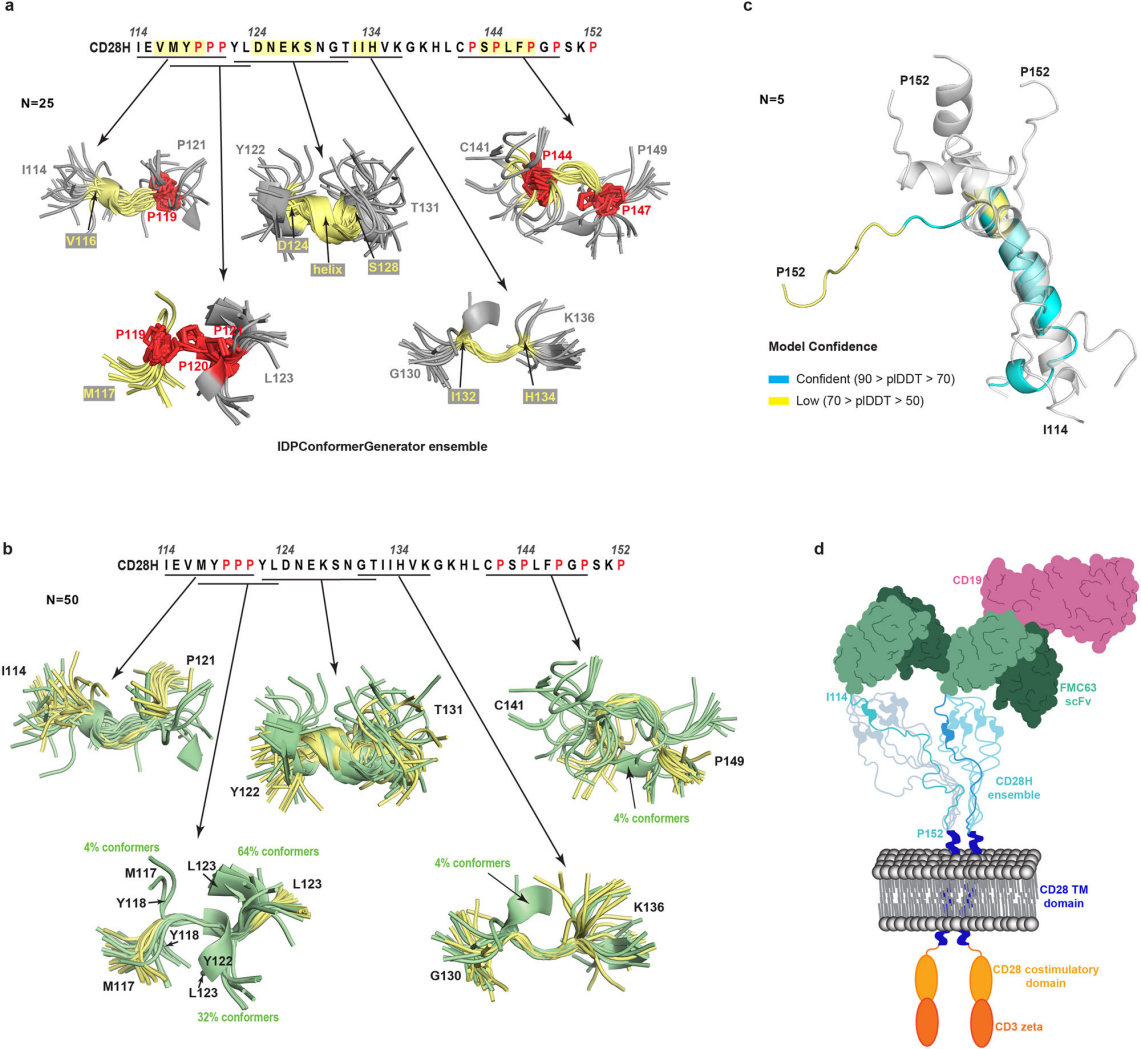
Comparison of the experimentally determined structural ensemble for CD28H with structures predicted by IDPConformerGenerator and AlphaFold 3. **a** PyMOL cartoon representation of structures aligned as in Fig. 3d for the CD28H ensemble generated by IDPConformerGenerator^40^ highlighting portions that exhibit local structure (yellow) and prolines (red). Structures are colored as in Fig. 3d. **b** CD28H structures predicted by IDPConformerGenerator (green, 25 structures) aligned to the structures calculated by using NMR data (yellow, 25 structures) for the regions shown in **a**. The percentage of conformers with long-range contacts that are not consistence with NOE data are indicated for the regions spanning G130 – K136 and C141 – P149. **c** Ribbon diagrams of five aligned AlphaFold 3-predicted models of CD28H. One model is colored according to the AlphaFold 3 per-residue confidence score (pLDDT) as indicated, whereas the other models are transparent and colored grey. **d** Schematic of an axicabtagene ciloleucel dimer bound to CD19. Each domain of the CAR is colored as in Fig. 1a, with the CD19 antigen colored pink. The CD28H is depicted as a globally disordered structural ensemble in the background, and the hinge domains of two CAR constructs are highlighted in the forefront.

In our study, we used NMR data in Xplor-NIH 3.7 to calculate a structural ensemble for CD28H. Collecting NOESY data was key to studying the local structures present in CD28H, which otherwise we would not have been detected. The software platform IDPConformerGenerator was developed to predict structures of intrinsically disordered proteins based on protein structures in the Protein Data Bank^40^. We inputted the CD28H sequence into IDPConformerGenerator to find similarities in the predicted ensemble compared to the Xplor-NIH structures. In particular, the predicted ensemble showed convergence for the regions spanning V116 – P119, P119 – P121, D124 – S128, I132 – H134, and P144 – P147 (Fig. 7a), with similar structural features to those observed experimentally (Fig. 3d). Some notable differences are observed, however. The NMR data support a 3_10_-helix for D124-S128 (Fig. 3e), as no NOE interactions are observed from Hα atoms to amide groups of residues C-terminal by four amino acids (Fig. 3b). IDPConformerGenerator predicts helicity in this region (Fig. 7a), but with 3_10_ (Supplementary Fig. 7a) and α helical (Supplementary Fig. 7b) configurations (Supplementary Fig. 7a, b). Moreover, in the region spanning P119 – P121, IDPConformerGenerator predicted 36% of conformers to form long-range contacts between M117/Y118 and Y122/L123 (Fig. 7b). Such contacts are not supported by NOE data (Fig. 3b). We also evaluated predictions from AlphaFold 3 for CD28H to find 60% of the outputted structures were predicted to be helical in the region spanning L123 – K138 (Fig. 7c). Thus, the predictions from IDPConformerGenerator more closely matched our experimental data. Our findings imply that IDPConformerGenerator can offer reasonable predictions for intrinsically disordered regions and moreover, that local structural features can be experimentally detected by using NOESY data.

In summary, we find CD28H to adopt local structural elements and undergo dynamic exchange between different configurations that include cis-trans proline isomerization. These properties resemble those of the CD8α hinge^18^, suggesting that dynamics and local structure may play a role in CAR T-cell signaling. In the context of CD28H, we also find a reconfiguration of a β-strand into a 3_10_-helix based on the omission of amino acids that form inter-strand interactions. Formation of the local structures observed in CD28H may be associated with recognition events, as intrinsically disordered proteins have previously been reported to adopt local structural elements in complexes with binding partners^23,41,42^. It is possible that these structures in the intrinsically disordered proteins are selected from transiently populated structures present in the free protein. Such flipping of secondary structure based on context has important implications for structural prediction algorithms, and even single amino acid substitutions can stymie structural prediction by AlphaFold2^43^. The intrinsic dynamics and resulting heterogeneity of CARs make detailed structural study of the intact CAR T-cell/tumor cell interaction challenging. A common feature of this intercellular interaction is induced dimerization at each end by the transmembrane domain and antigen recognition domain, adding a key structural restraint on the hinge region^21,44^ (Fig. 7d). Modulation of the intermembrane distance between CAR T-cell and tumor cells has been linked to CAR T-cell killing efficacy^13,45^. We find the structure of CD28H to greatly differ from the hinge region in the context of native CD28. This difference is important to the CAR T-cell because CD28H loses the highly structured β-strand observed in CD28 and instead, forms a 3_10_-helix within its N-terminal portion. This structural difference may affect the spacing imposed by the hinge on the transmembrane domain relative to the antigen recognition domain. Future studies taking advantage of cryo-electron tomography, aimed at dissecting the importance of global structural features for CAR T-cell signaling in combination with local structural data, such as that which we have obtained for CD28H, is likely to inform the development of new CAR-based immunotherapies.

## Methods

### Protein sample preparation

CD28H (I114 – P152) was subcloned into the pGEX-6P-1 vector between BamHI and XhoI restriction sites in a frame with an N-terminal glutathione S-transferase (GST) tag and a PreScission protease cleavage site. The construct was purchased through GenScript and contains codons optimized for expression in *Escherichia coli*. The plasmid was transformed into *Escherichia coli* strain BL21 (DE3) (Thermo Fisher Scientific C600003) with selection by ampicillin. The transformed colonies were grown in 10 mL of Luria-Bertani Broth (LB) medium (ampicillin 100 μg/ml) overnight at 37 °C with shaking and centrifuged for 10 minutes at 2000 g. Bacterial pellets containing CD28H construct were gently resuspended and diluted at 1:100 ratio into 1 L of M9 minimal media supplemented with 1 g/L ^15^N ammonium chloride (Sigma-Aldrich) and 3 g/L ^13^C glucose (Sigma-Aldrich) as the sole nitrogen or carbon source respectively. Cells were grown at 37 °C with shaking until they reached an OD_600_ of 0.5-0.6 at which point isopropyl β-D-1-thiogalactopyranoside (UBPBio) was added to a final concentration of 0.4 mM to induce protein expression at 17 °C overnight. The cells were pelleted by centrifugation at 4000 rpm and 4 °C for 45 minutes by using a Beckman Coulter J6-M1 centrifuge with a JS-4.2 rotor and then stored at −80 °C until purification.

Following resuspension in buffer 1 (20 mM HEPES at pH 7.5, 300 mM NaCl, 2 mM dithiothreitol, and an EDTA-free protease inhibitor cocktail tablet (Roche Diagnostics 11836170001)), cells were lysed via sonication and centrifuged at 27,000 *g* and 4 °C for 30 minutes. The supernatant was incubated with pre-washed glutathione sepharose beads (Cytiva 17-0756-05) for three hours at 4 °C. The beads were then washed extensively with buffer 1. Additional washes were conducted using buffer 2 (20 mM HEPES at pH 7.0, 50 mM NaCl, and 2 mM DTT). CD28H was cleaved from the GST tag by incubation overnight with PreScission protease. GST-tagged CD28H was eluted in buffer 2 with the addition of 20 mM reduced L-glutathione. CD28H was further purified by size exclusion chromatography (SEC) on an ÄKTA pure FPLC system (Cytiva) using a HiLoad 16/600 Superdex 75 prep grade column in buffer 3 (20 mM NaPO_4_ at pH 6.5, 50 mM NaCl, 2 mM DTT, and 20 μM ZnSO_4_) supplemented with an EDTA-free protease inhibitor cocktail tablet. GST-tagged CD28H was similarly purified by SEC in buffer 4 (20 mM HEPES pH 7.5, 50 mM NaCl, 2 mM DTT, 20 μM ZnSO_4_, EDTA-free protease inhibitor).

### SDS-PAGE

Protein lysates were subjected to SDS-PAGE on 12% NuPAGE Bis-Tris gels (Thermo Fisher Scientific NP0342) with MES SDS running buffer (Thermo Fisher Scientific NP0002) and visualized by Coomassie staining.

### Electrospray ionization mass spectrometry

Mass spectrometry was performed with CD28H (10 μM) in buffer 3 and GST-tagged CD28H (2 μM) in buffer 4 with 10% acetonitrile on a 6100 Series Quadrupole LC mass spectrometer (Agilent Technologies, Inc.), equipped with an electrospray source and operated in the positive ion mode. Data acquisition and analyses were performed using OpenLAB CDS ChemStation Edition software (version C.01.05, Agilent Technologies, Inc.).

### NMR samples and experiments

Five NMR samples were prepared including ^15^N, ^13^C-labeled CD28H at 500 μM (sample 1), 600 μM (sample 2), 560 μM (sample 3), 230 μM (sample 4), or 450 μM (sample 5) and ^15^N-labeled CD28H at 400 μM (sample 6), 280 μM (sample 7), 100 μM (sample 8), or 100 μM with MSPΔH5 nanodiscs (sample 9). 2D ^1^H-^15^N HSQC, 2D CON, 3D HNCACB/CBCA(CO)NH, 3D HNCO/HN(CA)CO, and 3D HACAN spectra were collected on sample 1. 2D ^1^H-^13^C HSQC, 3D ^13^C-dispersed NOESY (120 ms mixing time), 3D CCH-TOCSY, and 3D HCCH-TOCSY spectra were collected on sample 2. 3D ^13^C-dispersed NOESY (250 ms mixing time) spectra were collected on sample 3. 3D long-range HNCO-COSY spectra were collected on sample 4. 2D CACO and 3D CANCO spectra were collected on sample 5. 3D ^15^N-dispersed NOESY (120 ms mixing time) spectrum was collected on sample 6. 2D ^1^H-^15^N HSQC and 3D HNHB spectra were collected on sample 7. 2D ^1^H-^15^N HSQC spectra were collected on samples 8 and 9. The mixing time of the 3D ^13^C-dispersed NOESY spectrum was optimized for sample 2 using 2D ^1^H, ^1^H planes with mixing times varying from 80 to 300 ms (Supplementary Fig. 8).

All experiments were collected in buffer 3 (20 mM NaPO_4_ at pH 6.5, 50 mM NaCl, 2 mM DTT, 20 μM ZnSO_4_) with 1 mM pefabloc, 0.1% NaN_3_, and 5% ^2^H_2_O / 95% ^1^H_2_O, except for the 2D ^1^H-^13^C HSQC, 3D ^13^C-dispersed NOESY, 3D CCH-TOCSY, and 3D HCCH-TOCSY experiments, which were collected on samples that were lyophilized in buffer 3 containing 1 mM pefabloc and 0.1% NaN_3_, and resuspended in 100% ^2^H_2_O. All experiments were collected at 25°C except for a 3D ^13^C-dispersed NOESY (250 ms mixing time), 3D ^15^N-dispersed NOESY (120 ms mixing time), 3D HNCO/HN(CA)CO, 3D HNCACB/CBCA(CO)NH, 2D and 3D long-range HNCO-COSY spectra, which were acquired at 11°C. The spectra were recorded on Bruker AvanceIII 700, 800, 850, or 900 MHz spectrometers equipped with cryogenically cooled probes and operating with TopSpin 3.6.

IPAP processing was applied to CON, CANCO, and CACO experiments in TopSpin prior to further processing. All NMR data processing was performed using NMRpipe^46^ and spectra were visualized and analyzed with XEASY^47^. Backbone ϕ and ψ torsion angle restraints were assessed by the TALOS+ program using HN, Hα, Cα, Hβ, C’, and N chemical shifts^48^. The structure of CD28H was calculated with Xplor-NIH 3.7^32^ using NOE and hydrogen bond distance restraints along with backbone ϕ and ψ torsion angle restraints determined using TALOS+. The structural refinement used an extended initial structure generated by Xplor-NIH^32^ and included TorsionDB and HBPot energy terms. Hydrogen bond restraints were included only in the final rounds of structure calculations. The major conformational state consisting of all trans prolines was used for the structure calculations. Distance restraints were obtained by integrating the intensities of the NOE signals by using the peakint script in XEASY^47^. All detected NOE signals were able to be assigned and were used in the structure calculations. Center averaging was used to define distances involving non-stereospecifically assigned methyl, methylene, and aromatic atoms, with a pseudo-atom correction applied^32,49^. Calibration of the ^15^N-dispersed NOESY experiment was done by assigning the average intra-residue HN to Hα NOE for residues E115 – Y118, Y122 – E126, and N129 to a distance of 3 Å. The ^13^C-edited NOESY experiment was similarly calibrated by assigning a 3 Å distance to the average of the intra-residue Cβ to Cδ NOE of residues P119 – P121, P142, and P144 and Cβ to Cγ NOEs of residues P119 – P121, P142, P144, P147, P149, and the proline residue from the non-native amino acid sequence GPLGS that remains following cleavage of the GST tag. The distance restraints (in Å) for the other NOE signals were determined relative to the 3 Å-associated NOEs by using the ratio 1/r^6^ with minimum van der Waals distances and maximum distance of 3.0, 3.5, 4.5, 5.5, or 6.5 Å. Due to steric clashes or violations of the distance restraints determined from NOE data, 12% (6 of 50) of the structures calculated by Xplor-NIH were excluded from the study, and the 25 lowest energy structures were chosen for visualization and statistical analyses. PyMOL (PyMOL Molecular Graphics System, https://www.pymol.org) was used to visualize structures, generate figures, and calculate their radii of gyration^50^ and torsion angles. Chemical shift index (CSI) values for CD28H Cα atoms were calculated by comparison to random coil values of the same amino acid type with sequence-dependent correction factors^36,37^. A CD28H structural ensemble was generated by inputting the CD28H sequence into the software IDPConformerGenerator^40^ in NMRbox (https://nmrbox.nmrhub.org)^51^.

### GST pull-down analysis

10 nmol of purified GST-tagged CD28H or GST (Fisher Scientific LLC, PI20237), as a negative control, were bound to 40 μL of prewashed glutathione sepharose resin and incubated with 20 nmol CD28H. Unbound protein was removed by extensive washing in buffer 4 (20 mM HEPES pH 7.5, 50 mM NaCl, 2 mM DTT, 20 μM ZnSO_4_, EDTA-free protease inhibitor). Proteins that were retained on the resin were resolved by SDS-PAGE.

### CD experiments

Far-UV range CD spectra of CD28H were recorded in buffer 5 (20 mM NaPO4 at pH 6.5, 10 mM NaCl, and 1 mM 2‑mercaptoethanol) on a Jasco J-1500CD spectrometer using a quartz cuvette with 1.0 mm path length. Thermostability measurements were collected for wavelengths 190 – 240 nm on CD28H (30 μM) following one minute of incubation at temperatures ranging from 25 °C to 80 °C. To collect secondary structure information, additional CD spectra were recorded in the far-UV range (190 – 250 nm) on CD28H (10 μM) with temperature controlled at 25 ± 0.1 °C. Buffer 5 was used as a control. All spectra were collected continuously at a scan speed of 20 nm/min and averaged over accumulation of three spectra. The buffer spectrum was subtracted from the protein spectra during data analyses. Molar ellipticity θ (in deg cm^2^ dmol^−1^) was calculated from the measured machine units m° in millidegrees at wavelength λ using Eq. (1).

1 $$\theta =\frac{{{\rm{m}}}^{\circ }}{(10\cdot C\cdot L)}\,$$

C is the concentration of the sample in mol$\cdot$L^-1^ and L is the path length of the cell (cm). Secondary structure analysis was conducted with the program CONTIN^52^ by the DichroWeb server^31^ using reference dataset SP175t (190–240 nm)^52^.

### AlphaFold 3 prediction

AlphaFold 3 prediction was utilized through the AlphaFold Server (https://alphafoldserver.com)^19^. Structures were analyzed and figures generated by using PyMOL (PyMOL Molecular Graphics System, http://www.pymol.org).

### Statistics and reproducibility

To calculate the protein structure, 50 random linear structures were used as starting structures. After simulated annealing and energy minimization steps, the 25 lowest energy structures were chosen for visualization and statistical analyses. The violations and deviations from idealized geometry in Table 1 were obtained by XPLOR-NIH and average pairwise root-mean-square deviation was calculated by MOLMOL. Mean values, standard deviation, and standard error were calculated by using Microsoft Excel. OpenLAB CDS ChemStation Edition software (version C.01.05, Agilent Technologies, Inc.) was used to deconvolute the mass spectrum in Fig. 1e. Biochemical experiments, including protein purification and SDS-PAGE were done in biological replicates of greater than 10 times. CD experiments were done in triplicate. 2D NMR, LC-MS, and AlphaFold 3 prediction were repeated at least once. All replications were consistent.

### Reporting summary

Further information on research design is available in the Nature Portfolio Reporting Summary linked to this article.

### Supplementary information

Peer Review File

Supplementary Information

Description of Additional Supplementary Files

Supplementary Data 1

Reporting summary

## Data Availability

Atomic coordinates for CD28H have been deposited in the Protein Data Bank (PDB) with accession number 8W2V. Chemical shift assignments have been deposited in the Biological Magnetic Resonance Data Bank (BMRB) with accession number 31150. Plasmid pGEX-6P-1-CD28 CAR hinge (I114-P142) was deposited in Addgene with ID 224929. Source data are provided for this manuscript in Supplementary Data 1. Uncropped gel images for Fig. 1f and Supplementary Fig. 1 are included as Supplementary Fig. 9 and 10 respectively in the Supplementary Information. All other data are available from the corresponding author on reasonable request.
