# Supplementary material for: CD28 hinge used in chimeric antigen receptor (CAR) T-cells exhibits local structure and conformational exchange amidst global disorder
Source: Commun Biol. 2024 Aug 31;7:1072. doi: 10.1038/s42003-024-06770-w (PMC11365992; doi:10.1038/s42003-024-06770-w)
Supplement: Supplementary file 2 — Supplementary Information [file 42003_2024_6770_MOESM2_ESM.pdf]

Supplementary Information

CD28 hinge used in chimeric antigen receptor (CAR) T-cells exhibits local structure and conformational exchange amidst global disorder

Varvara Folimonova[1], Xiang Chen[1], Hitendra Negi[1], Charles D. Schwieters[2], Jess Li[3], R. Andrew Byrd[3], Naomi Taylor[4], Philippe Youkharibache[5], Kylie J. Walters[1,*]

[1]Protein Processing Section, Center for Structural Biology, Center for Cancer Research, National Cancer Institute, National Institutes of Health, Frederick, MD 21702, USA
[2]Computational Biomolecular Magnetic Resonance Core, National Institute of Diabetes and Digestive and Kidney Diseases, NIH, Bethesda, MD 20892, USA
[3]Structural Biophysics Laboratory, Center for Cancer Research, National Cancer Institute, Frederick, MD 21702, USA
[4]Pediatric Oncology Branch, Center for Cancer Research, National Cancer Institute, National Institutes of Health, Bethesda, MD 20814, USA
[5]Cancer Data Science Laboratory, Center for Cancer Research, National Cancer Institute, National Institutes of Health, Bethesda, MD 20894, USA

Supplementary Figure 1: ^{15}N-labeled CD28H used for NMR is pure following size exclusion chromatography.

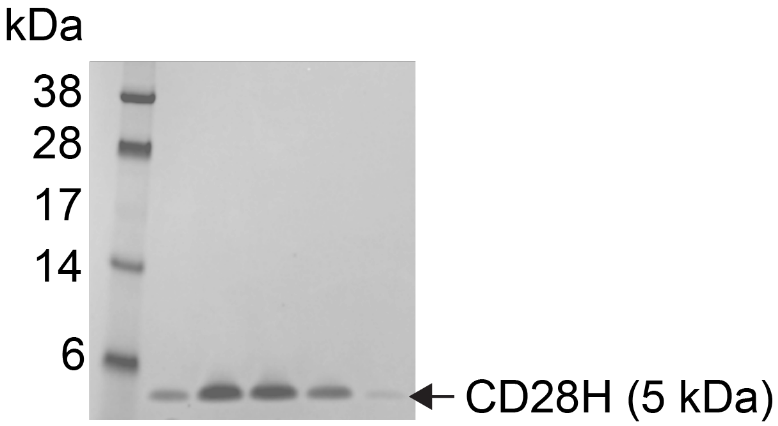

SDS-PAGE following size exclusion chromatography of ^{15}N-labeled CD28H. The sample was loaded onto Superdex 75 column on an FPLC system. A standard molecular weight marker is included in the left-most lane.

Supplementary Figure 2: CD28H exhibits multiple conformations in NMR spectra.

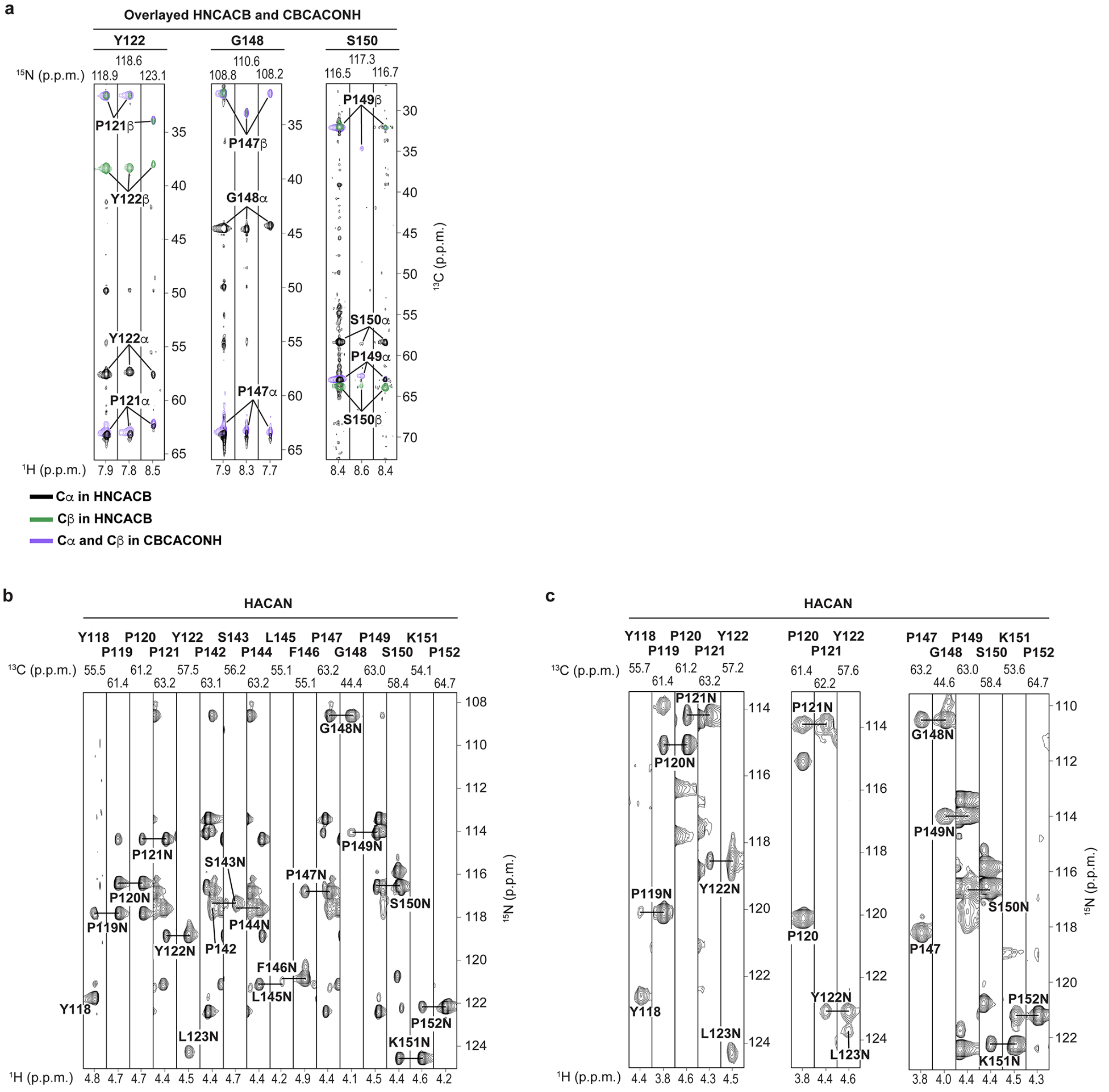

a Overlayed strip plots of the 2D ^{1}H, ^{13}C planes from HNCACB (Cα signals in black and Cβ signals in green) and CBCACONH (Cα and Cβ signals in purple) spectra, illustrating residues with three sets of resonances. **b** Strip plots of the 2D ^{1}H, ^{15}N plane from a 3D HACAN experiment, showing signals from residues Y118 – Y122 and P142 – P152 associated with the major conformational state of CD28H. **c** 2D ^{1}H, ^{15}N strip plots from the 3D HACAN experiment, showing signals associated with minor conformational states of CD28H Y118 – Y122 (left), P120 – Y122 (center), and P147 – P152 (right). For all experiments, a sample of 500 μM ^{15}N, ^{13}C-labeled CD28H was used and data collected at 25°C on a spectrometer operating at 800 MHz equipped with a cryogenically cooled probe.

Supplementary Figure 3: Multiple signals are observed for S143 and L145 whereas P144 exhibits only one set of signals.

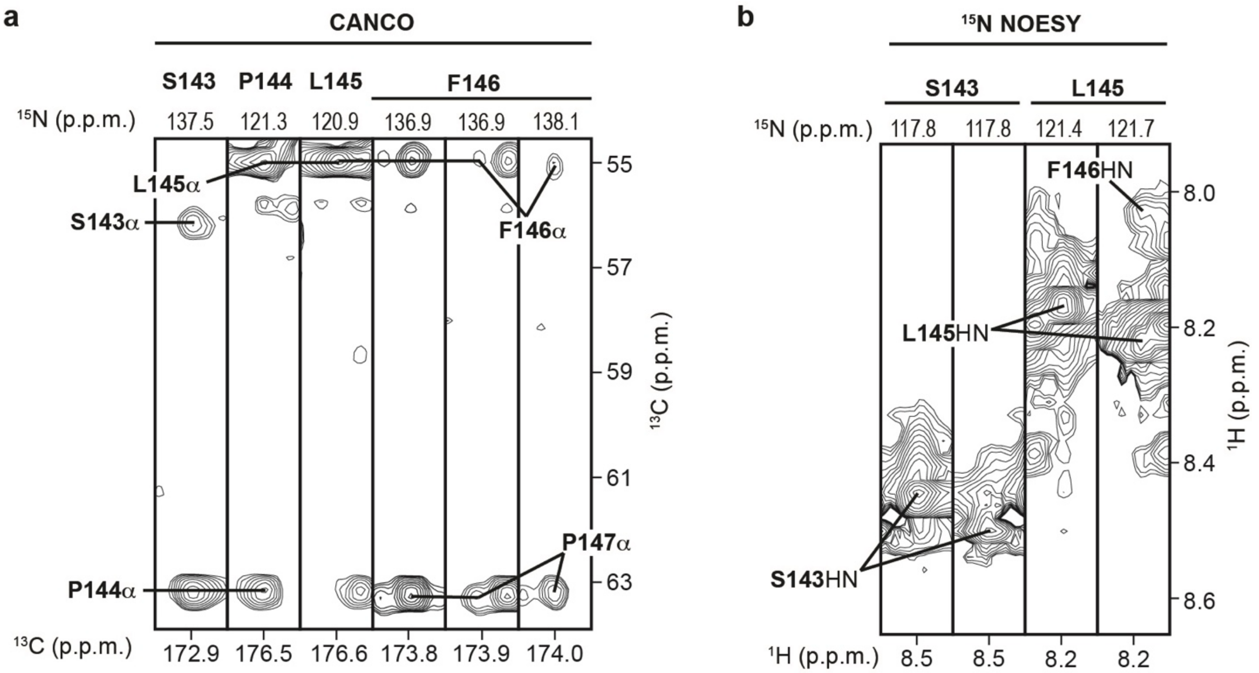

a Strip plots of the 2D ^{13}C, ^{13}C planes from CANCO spectrum, showing convergence of S143, P144, and L145 signals. The CANCO spectrum was collected on 450 μM ^{15}N, ^{13}C CD28H at 25°C on a spectrometer operating at 700 MHz equipped with a cryogenically cooled probe. **b** Strip plots of 2D ^{1}H, ^{1}H planes from ^{15}N-dispersed NOESY experiment recorded at 800 MHz on 400 μM ^{15}N CD28H at 11°C. S143 and L145 show two conformational states.

Supplementary Figure 4: Removal of dithiothreitol (DTT) or inclusion of $ZnSO_4$ does not cause signal shifting in an ^{1}H, ^{15}N HSQC spectrum of CD28H.

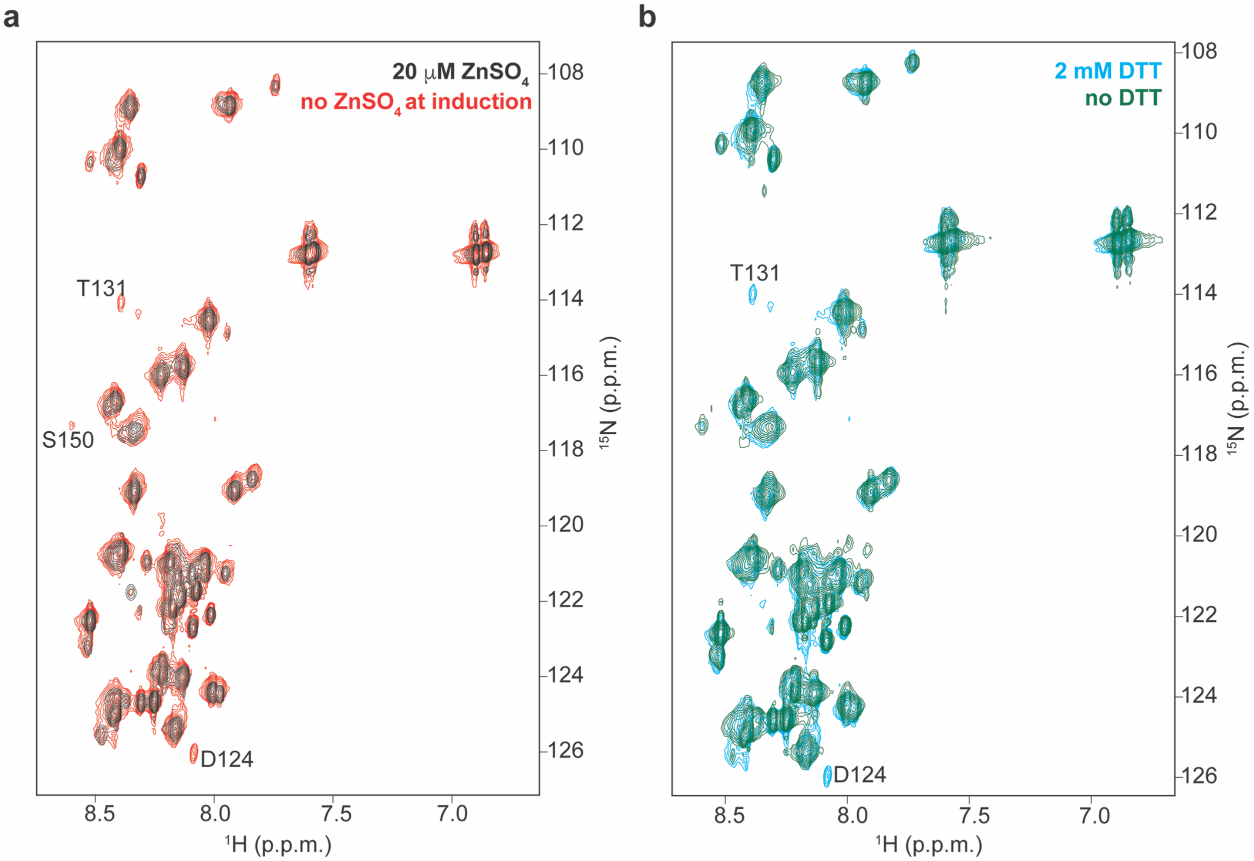

a Superimposed ^{1}H, ^{15}N HSQC spectra of 500 µM ^{15}N, ^{13}C-labeled CD28H without 20 µM $ZnSO_4$ (red) or 280 µM ^{15}N-labeled CD28H expressed with 20 µM $ZnSO_4$ added at induction (black). **b** Superimposed ^{1}H, ^{15}N HSQC spectra of 500 µM ^{15}N, ^{13}C-labeled CD28H with 2 mM DTT (blue) or 450 µM ^{15}N-labeled CD28H without DTT (green). Signals that appear only for the sample prepared without $ZnSO_4$ included at induction (**a**) or with 2 mM DTT (**b**) are labeled with their assignment. The spectra were recorded at 25°C and 800 MHz (without $ZnSO_4$ added at induction, and with 2 mM DTT), 700 MHz (with $ZnSO_4$ added at induction), and 850 MHz (without DTT). Except for the sample without DTT, all samples were dissolved in a buffer composed of 20 mM $NaPO_4$ at pH 6.5, 50 mM NaCl, 2 mM DTT, 20 µM $ZnSO_4$, 1 mM pefabloc, 0.1% NaN_3, and 5% ^2H_2O / 95% ^1H_2O.

Supplementary Figure 5: Selected regions of 2D or 3D long-range HNCO-COSY spectra acquired on CD28H.

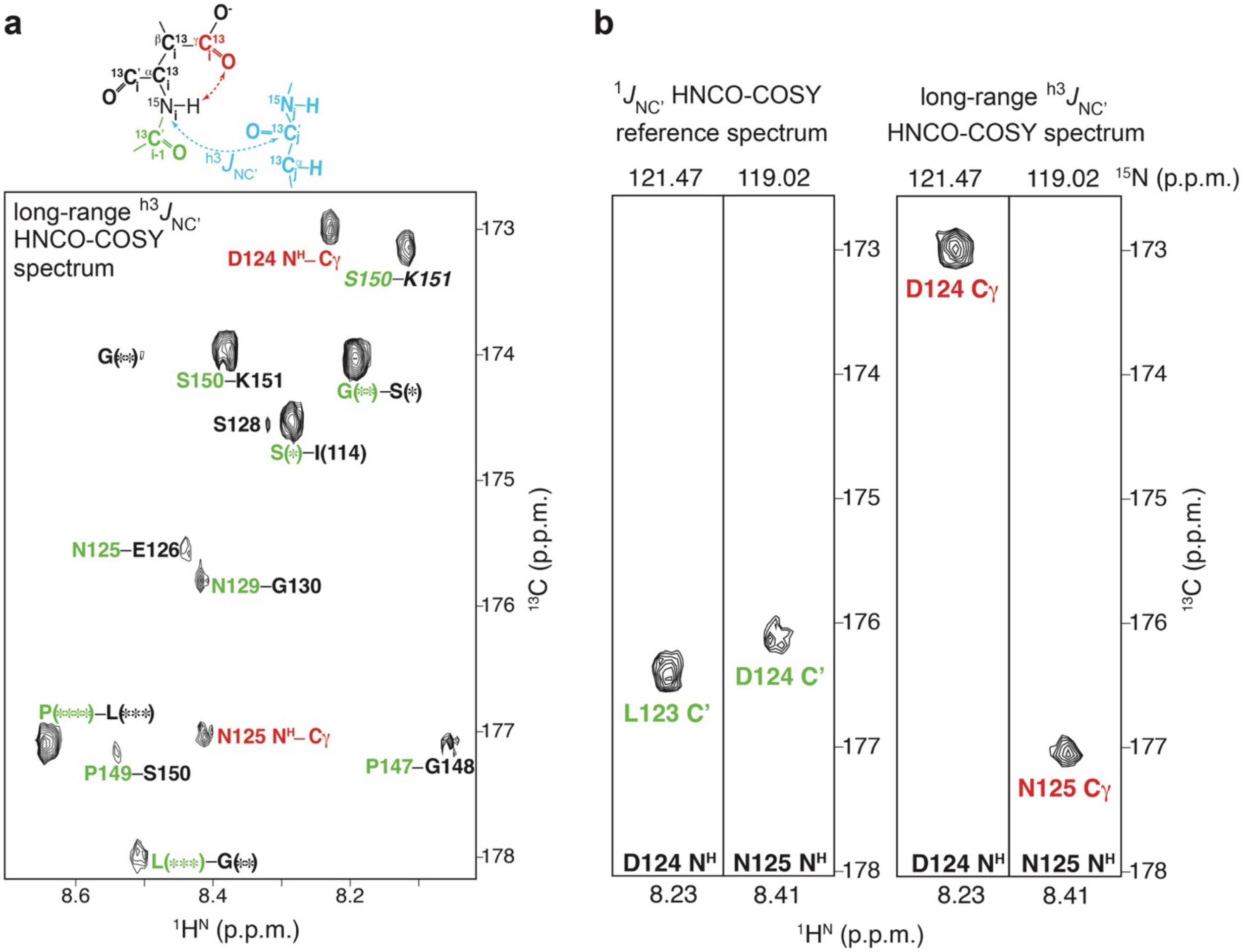

a Selected region of a 2D long-range HNCO-COSY spectrum acquired on 230 μM ^{15}N, ^{13}C-labeled CD28H collected at 850 MHz and 11°C. Intra-residue $^{h3}J_{NC'}$ hydrogen bonds between the backbone amide atoms of D124 and N125 their respective side chain oxygen atoms are labeled in red. Incompletely suppressed sequential correlations between the ^{15}N nucleus of residue i (black) and ^{13}C' nucleus of residue i-1 (green) are labeled. Signals from the non-native amino acid sequence LGS that remains following cleavage of the GST tag are labeled by three (***), two (**), or one (*) asterisk, respectively. **b** Strip plots of a 3D $^1J_{NC'}$ reference spectrum (left) and 3D long-range $^{h3}J_{NC'}$ HNCO-COSY spectrum (right) acquired as in **a**. Sequential (green) and intra-residue H-bond (red) correlations between carbonyls and backbone amide protons are marked with assignment information.

Supplementary Figure 6: Inclusion of MSPΔH5 nanodiscs does not cause spectra changes in an ^{1}H, ^{15}N HSQC spectrum of CD28H.

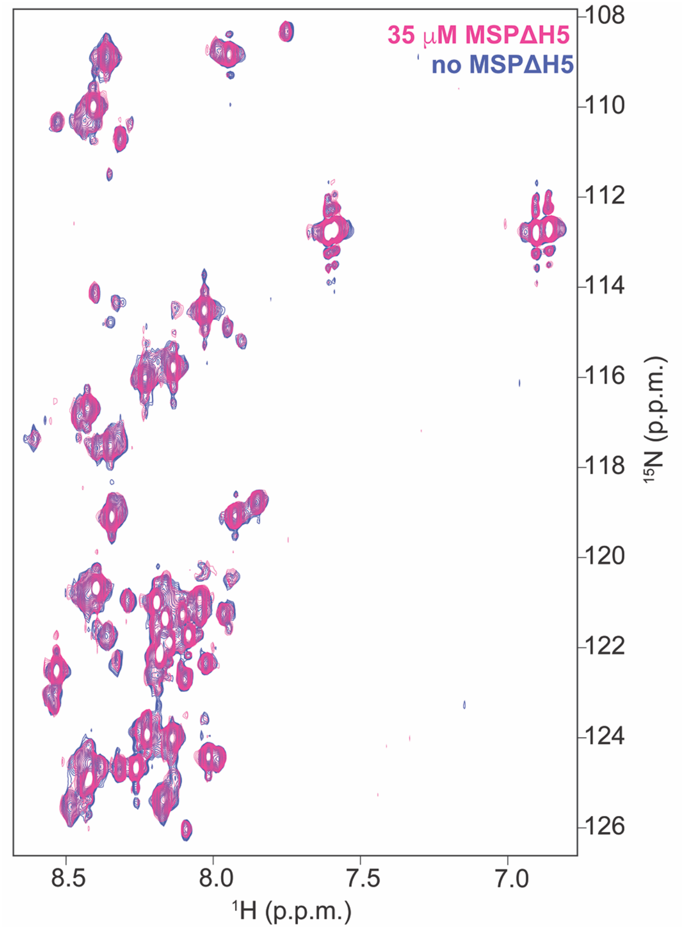

Superimposed ^{1}H, ^{15}N HSQC spectra of 100 μM ^{15}N-labeled CD28H with (pink) or without (violet) 35 μM MSPΔH5 nanodiscs. The spectra were recorded at 25°C and 800 MHz. Samples were dissolved in a buffer composed of 20 mM $NaPO_4$ at pH 6.5, 50 mM NaCl, 2 mM DTT, 20 μM $ZnSO_4$, 1 mM pefabloc, 0.1% NaN_3, and 5% $^{2}H_2O$ / 95% $^{1}H_2O$.

Supplementary Figure 7: IDPConformerGenerator predicts both a 3_{10}-helix and α-helix configuration for the region spanning D124 – S128.

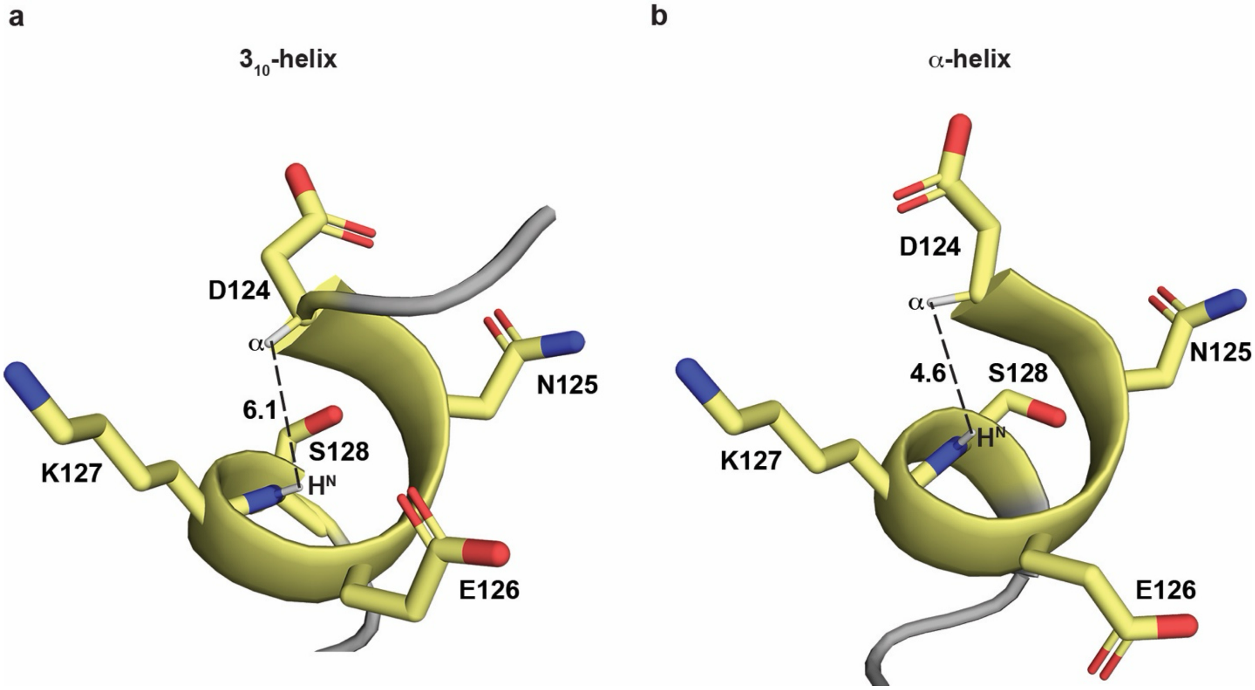

PyMOL cartoon structure for CD28H D124 – S128 highlighting interactions of the predicted 3_{10}-helix (a) or α-helix (b) with heavy sidechain atoms displayed as sticks and oxygen (with double or single valency indicated), nitrogen, and select hydrogen atoms colored red, indigo and white, respectively. Distances (in Å) between D124 Hα and S128 H^N are indicated and displayed by black dashed lines.

Supplementary Figure 8: 3D ^{13}C-dispersed NOESY experiment acquired on CD28H with varying mixing times.

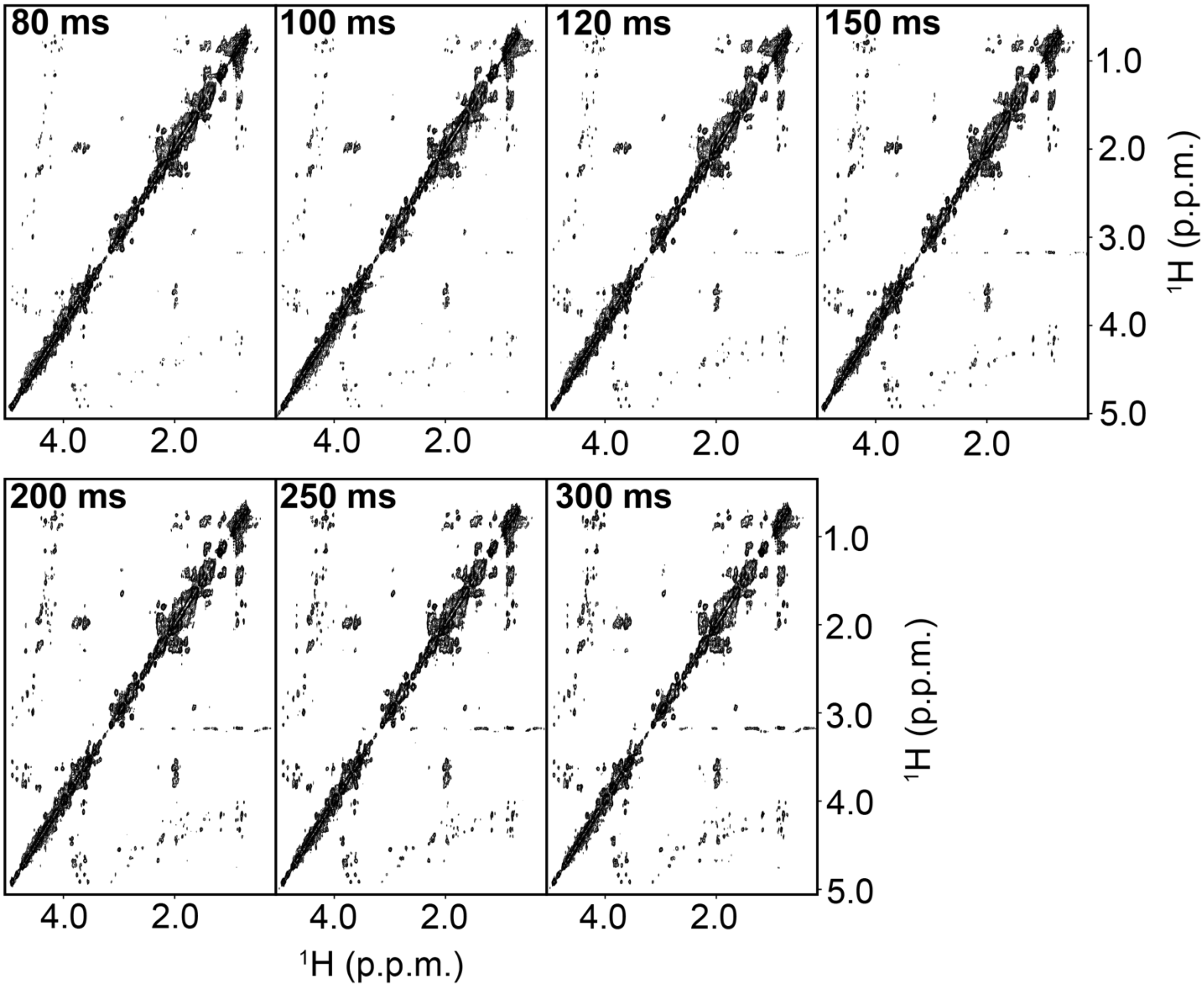

2D ^{1}H, ^{1}H planes of a ^{13}C-dispered NOESY experiment with mixing times of 80, 100, 120, and 150 ms (top, from left to right) and of 200, 250, and 300 ms (bottom, from left to right). Data were collected on a sample of 600 μM ^{15}N, ^{13}C-labeled CD28H at 25°C on a spectrometer operating at 800 MHz.

Supplementary Figure 9: Uncropped gel image of Fig. 1f.

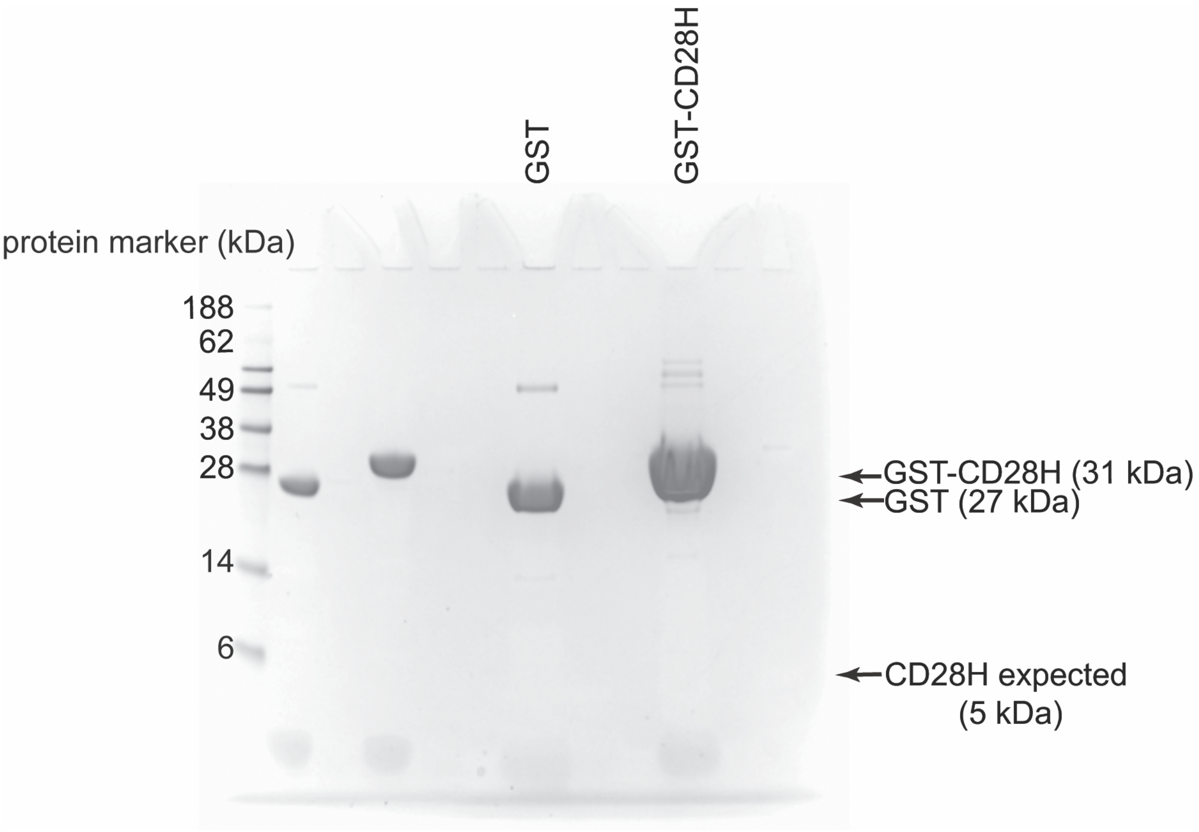

GST pull-down indicates that CD28H does not form oligomers. CD28H was incubated with glutathione beads prebound with GST (left, 27 kDa) or GST-tagged CD28H (right, 31 kDa), unbound proteins removed by washing the beads, and those retained on the resin observed by SDS-PAGE. The expected position of CD28H (5 kDa) is indicated.

Supplementary Figure 10: Uncropped gel image of Supplementary Figure 1.

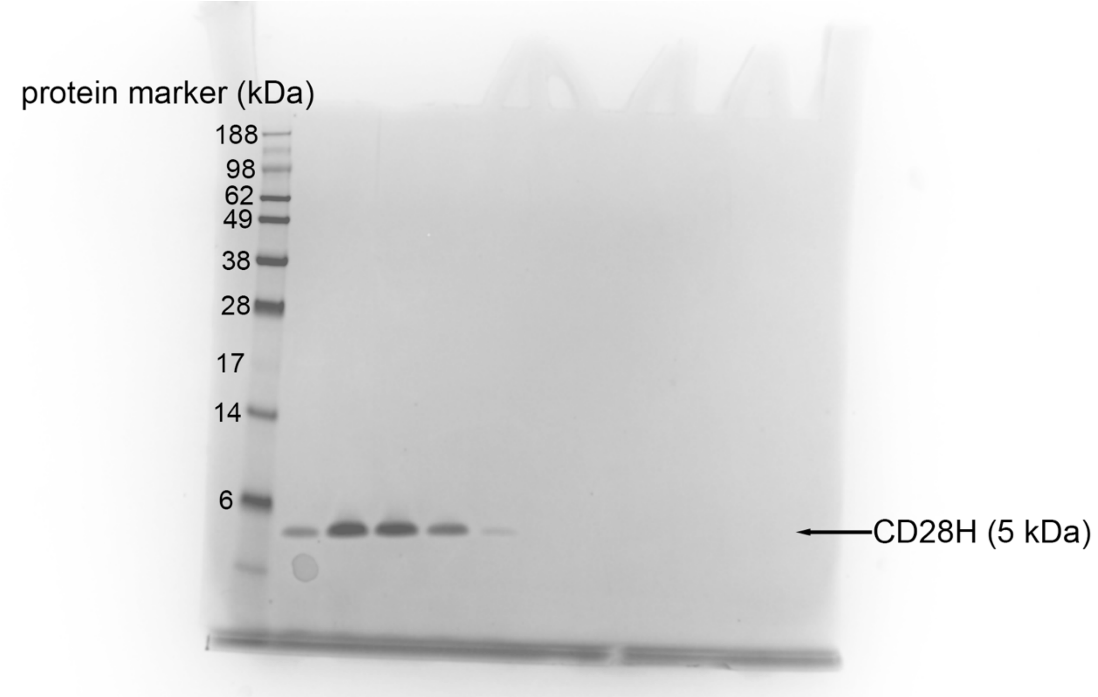

SDS-PAGE of ^{15}N-labeled CD28H fractions from size exclusion chromatography with a Superdex 75 column on an FPLC system. A standard molecular weight marker is included in the left-most lane.

Supplementary Table 1. Chemical shift values of HN resonances in CD28H. The HN chemical shift of some minor conformers were not determined and are indicated with N.D.

	Major	Minor states	
**I114**	8.261		
**E115**	8.468	8.485	
**V116**	8.193	8.273	N.D.
**M117**	8.299	8.405	N.D.
**Y118**	8.251	8.172	8.131
**Y122**	8.085	8.014	8.586
**L123**	8.084	8.051	8.085
**D124**	8.206		8.350
**N125**	8.389		
**E126**	8.413		
**K127**	8.235		
**S128**	8.289		
**N129**	8.477		
**G130**	8.398		
**T131**	8.073		
**I132**	8.211		
**I133**	8.261		
**H134**	8.539		
**V135**	8.316		
**K136**	8.563		
**G137**	8.523		
**K138**	8.224		
**H139**	8.502		
**L140**	8.303		
**C141**	8.447		
**S143**	8.451	8.499	
**L145**	8.178	7.787	
**F146**	8.118	8.044	N.D.
**G148**	8.036	8.389	7.816
**S150**	8.509	N.D.	8.536
**K151**	8.357	N.D.	8.101
