# Supplementary material for: CD28 hinge used in chimeric antigen receptor (CAR) T-cells exhibits local structure and conformational exchange amidst global disorder
Source: Commun Biol. 2024 Aug 31;7:1072. doi: 10.1038/s42003-024-06770-w (PMC11365992; doi:10.1038/s42003-024-06770-w)
Supplement: Supplementary file 5 — Reporting summary [file 42003_2024_6770_MOESM5_ESM.pdf]

nature portfolio

Corresponding author(s): Kylie Walters

Last updated by author(s): Aug 5, 2024

Reporting Summary

Nature Portfolio wishes to improve the reproducibility of the work that we publish. This form provides structure for consistency and transparency in reporting. For further information on Nature Portfolio policies, see our Editorial Policies and the Editorial Policy Checklist.

Statistics

For all statistical analyses, confirm that the following items are present in the figure legend, table legend, main text, or Methods section.

n/a	Confirmed	
☐	☒	The exact sample size (*n*) for each experimental group/condition, given as a discrete number and unit of measurement
☐	☒	A statement on whether measurements were taken from distinct samples or whether the same sample was measured repeatedly
☒	☐	The statistical test(s) used AND whether they are one- or two-sided<br>*Only common tests should be described solely by name; describe more complex techniques in the Methods section.*
☒	☐	A description of all covariates tested
☒	☐	A description of any assumptions or corrections, such as tests of normality and adjustment for multiple comparisons
☐	☒	A full description of the statistical parameters including central tendency (e.g. means) or other basic estimates (e.g. regression coefficient) AND variation (e.g. standard deviation) or associated estimates of uncertainty (e.g. confidence intervals)
☒	☐	For null hypothesis testing, the test statistic (e.g. *F*, *t*, *r*) with confidence intervals, effect sizes, degrees of freedom and *P* value noted<br>*Give P values as exact values whenever suitable.*
☒	☐	For Bayesian analysis, information on the choice of priors and Markov chain Monte Carlo settings
☒	☐	For hierarchical and complex designs, identification of the appropriate level for tests and full reporting of outcomes
☒	☐	Estimates of effect sizes (e.g. Cohen's *d*, Pearson's *r*), indicating how they were calculated

Our web collection on statistics for biologists contains articles on many of the points above.

Software and code

Policy information about availability of computer code

Data collection	Bruker TopSpin (version 3.6.5 and 3.7), 6100 Series Quadrupole LC mass spectrometer equipped with an electrospray source and operated in the positive ion mode.
Data analysis	NMRpipe (version 11.5), XEASY (version 1.3.11), TALOS+ (version 11.5), XPLOR-NIH (version 3.8), MOLMOL (version 2k2), PyMOL (version 2.5.7), OpenLAB CDS ChemStation Edition (version C.01.05), Microsoft Excel (version 16.86).

For manuscripts utilizing custom algorithms or software that are central to the research but not yet described in published literature, software must be made available to editors and reviewers. We strongly encourage code deposition in a community repository (e.g. GitHub). See the Nature Portfolio guidelines for submitting code & software for further information.

Data

Policy information about availability of data

All manuscripts must include a data availability statement. This statement should provide the following information, where applicable:

- Accession codes, unique identifiers, or web links for publicly available datasets
- A description of any restrictions on data availability
- For clinical datasets or third party data, please ensure that the statement adheres to our policy

Atomic coordinates for CD28H have been deposited in the Protein Data Bank (PDB) with accession number 8W2V. Chemical shift assignments have been deposited

in the Biological Magnetic Resonance Data Bank (BMRB) with accession number 31150. Plasmid pGEX-6P-1-CD28 CAR hinge (I114-P142) was deposited to Addgene with the ID 224929.

Research involving human participants, their data, or biological material

Policy information about studies with human participants or human data. See also policy information about sex, gender (identity/presentation), and sexual orientation and race, ethnicity and racism.

Reporting on sex and gender	N/A
Reporting on race, ethnicity, or other socially relevant groupings	N/A
Population characteristics	N/A
Recruitment	N/A
Ethics oversight	N/A

Note that full information on the approval of the study protocol must also be provided in the manuscript.

Field-specific reporting

Please select the one below that is the best fit for your research. If you are not sure, read the appropriate sections before making your selection.

☒ Life sciences ☐ Behavioural & social sciences ☐ Ecological, evolutionary & environmental sciences

For a reference copy of the document with all sections, see nature.com/documents/nr-reporting-summary-flat.pdf

Life sciences study design

All studies must disclose on these points even when the disclosure is negative.

Sample size	No statistical methods were applied to pre-determined sample sizes. During structure calculations of the protein complex, 50 random linear structures were used as starting structures. After simulated annealing and energy minimization steps, the 25 lowest energy structures were chosen for visualization and statistical analyses.
Data exclusions	Due to steric clashes or violations of the distance constraints determined from NOE data, 12% (6 of 50) of the structures calculated by Xplor-NIH were excluded from the study.
Replication	Biochemical experiments, including protein purification and SDS-PAGE were done in biological replicates of greater than 10 times. CD experiments were done in triplicate. 2D NMR, LC-MS and AlphaFold 3 prediction were repeated at least once. All replications were consistent.
Randomization	This study did not allocate experimental groups thus no randomization was required for the reported experiments.
Blinding	Structural determination and biophysical assays are used in this study. No human, animal or behavioral experiments were performed in this study, thus blinding was not required in this study. All experiments were done with appropriate positive or negative controls as indicated.

Reporting for specific materials, systems and methods

We require information from authors about some types of materials, experimental systems and methods used in many studies. Here, indicate whether each material, system or method listed is relevant to your study. If you are not sure if a list item applies to your research, read the appropriate section before selecting a response.

Materials & experimental systems

n/a	Involved in the study	
☒	☐	Antibodies
☒	☐	Eukaryotic cell lines
☒	☐	Palaeontology and archaeology
☒	☐	Animals and other organisms
☒	☐	Clinical data
☒	☐	Dual use research of concern
☒	☐	Plants

Methods

n/a	Involved in the study	
☒	☐	ChIP-seq
☒	☐	Flow cytometry
☒	☐	MRI-based neuroimaging

Plants

Seed stocks	N/A
Novel plant genotypes	N/A
Authentication	N/A
