# Supplementary material for: CD28 hinge used in chimeric antigen receptor (CAR) T-cells exhibits local structure and conformational exchange amidst global disorder
Source: Commun Biol. 2024 Aug 31;7:1072. doi: 10.1038/s42003-024-06770-w (PMC11365992; doi:10.1038/s42003-024-06770-w)
Supplement: Supplementary file 1 — Peer Review File [file 42003_2024_6770_MOESM1_ESM.pdf]

nature portfolio

Peer Review File

CD28 hinge used in chimeric antigen receptor (CAR) T-cells exhibits local structure and conformational exchange amidst global disorder

Open Access This file is licensed under a Creative Commons Attribution 4.0 International License, which permits use, sharing, adaptation, distribution and reproduction in any medium or format, as long as you give appropriate credit to the original author(s) and the source, provide a link to the Creative Commons license, and indicate if changes were made. In the cases where the authors are anonymous, such as is the case for the reports of anonymous peer reviewers, author attribution should be to 'Anonymous Referee' followed by a clear attribution to the source work. The images or other third party material in this file are included in the article's Creative Commons license, unless indicated otherwise in a credit line to the material. If material is not included in the article's Creative Commons license and your intended use is not permitted by statutory regulation or exceeds the permitted use, you will need to obtain permission directly from the copyright holder. To view a copy of this license, visit http://creativecommons.org/licenses/by/4.0/.

REVIEWERS' COMMENTS:

Reviewer #1 (Remarks to the Author):

While I appreciate some of additional clarifications made, I fear the manuscript still suffers from an abundance of technical detail in the text itself that highly field-specific and detracts from overall understanding of the experiments and broad points. Largely I still am unable to evaluate the science as this is not written a broad level, re-enforcing my suggestion that this be published in a field-specific and not general journal.

Reviewer #3 (Remarks to the Author):

The authors have prepared a revised version of the manuscript that takes into consideration the points that were raised by the reviewers. I recommend publication of the revised manuscript.
